# A microCT-based atlas of the central nervous system and midgut in sea spiders (Pycnogonida) sheds first light on evolutionary trends at the family level

**DOI:** 10.1186/s12983-022-00459-8

**Published:** 2022-03-31

**Authors:** Karina Frankowski, Katsumi Miyazaki, Georg Brenneis

**Affiliations:** 1 Zoologisches Institut und Museum, AG Cytologie und Evolutionsbiologie, Universität Greifswald, Soldmannstraße 23, 17489 Greifswald, Germany; 2 Department of Environmental Science, Faculty of Science, Niigata University, 8050 Ikarashi 2-no-cho, Niigata, 950-2181 Japan

**Keywords:** Chelicerata, Pantopoda, Neuroanatomy, Ventral nerve cord, Digestive system, Evolution, Phylogeny, 3D reconstruction, X-ray tomography

## Abstract

**Background:**

Pycnogonida (sea spiders) is the sister group of all other extant chelicerates (spiders, scorpions and relatives) and thus represents an important taxon to inform early chelicerate evolution. Notably, phylogenetic analyses have challenged traditional hypotheses on the relationships of the major pycnogonid lineages (families), indicating external morphological traits previously used to deduce inter-familial affinities to be highly homoplastic. This erodes some of the support for phylogenetic information content in external morphology and calls for the study of additional data classes to test and underpin in-group relationships advocated in molecular analyses. In this regard, pycnogonid internal anatomy remains largely unexplored and taxon coverage in the studies available is limited.

**Results:**

Based on micro-computed X-ray tomography and 3D reconstruction, we created a comprehensive atlas of in-situ representations of the central nervous system and midgut layout in all pycnogonid families. Beyond that, immunolabeling for tubulin and synapsin was used to reveal selected details of ganglionic architecture. The ventral nerve cord consistently features an array of separate ganglia, but some lineages exhibit extended composite ganglia, due to neuromere fusion. Further, inter-ganglionic distances and ganglion positions relative to segment borders vary, with an anterior shift in several families. Intersegmental nerves target longitudinal muscles and are lacking if the latter are reduced. Across families, the midgut displays linear leg diverticula. In Pycnogonidae, however, complex multi-branching diverticula occur, which may be evolutionarily correlated with a reduction of the heart.

**Conclusions:**

Several gross neuroanatomical features are linked to external morphology, including intersegmental nerve reduction in concert with trunk segment fusion, or antero-posterior ganglion shifts in partial correlation to trunk elongation/compaction. Mapping on a recent phylogenomic phylogeny shows disjunct distributions of these traits. Other characters show no such dependency and help to underpin closer affinities in sub-branches of the pycnogonid tree, as exemplified by the tripartite subesophageal ganglion of Pycnogonidae and Rhynchothoracidae. Building on this gross anatomical atlas, future studies should now aim to leverage the full potential of neuroanatomy for phylogenetic interrogation by deciphering pycnogonid nervous system architecture in more detail, given that pioneering work on neuron subsets revealed complex character sets with unequivocal homologies across some families.

**Supplementary Information:**

The online version contains supplementary material available at 10.1186/s12983-022-00459-8.

## Background

Pycnogonids (sea spiders) are marine arthropods with a cosmopolitan distribution in benthic habitats [1]. While some representatives in deep-sea and polar regions can reach considerable dimensions of up to 75 cm leg span [2], shallow water species are generally smaller-sized and often cryptic, as their coloration tends to blend in with the substrate they cling to [3]. The phylogenetic position of pycnogonids in the arthropod tree of life has been matter of considerable debate [4]. In the meantime, they are securely anchored in the Chelicerata (spiders, scorpions, horseshoe crabs and kin), where they form the sister group of all other extant lineages, the Euchelicerata [5–9].

Based on their unmistakable body organization, extant pycnogonids are instantly recognizable. They have a small, often tube-like body that is divided into an anterior cephalosoma and the trunk segments and is typically equipped with four pairs of prominent, nine-articled legs (in a few taxa five or six pairs) inserting on lateral segment processes. The cephalosoma is formed by four fused body segments and bears (1) a dorsal ocular tubercle typically equipped with two pairs of single-lensed eyes, (2) a suctorial proboscis composed of three longitudinal antimeres enclosing the Y-shaped pharynx in their center, (3) up to three cephalic limb pairs (the cheliphores, palps and ovigers) and (4) the first pair of legs. The remaining leg pairs are associated with the following trunk segments, of which the last one is additionally equipped with the posterior anal tubercle that represents an unsegmented remnant of the opisthosoma in other chelicerates [10, 11]. Remarkably, some or even all of the cephalic limbs can be variably reduced or completely missing in adults of different taxa (see [12]) and these differing limb configurations have provided important characters for the taxonomic classification of pycnogonids at the family and genus level [13–15]. However, contrary to the notion of a unidirectional gradual reduction of these limbs during pycnogonid evolution (e.g., [14, 16]), their variable presence and adult structure have been consistently indicated as highly homoplastic in phylogenetic analyses using external morphological [17] and molecular data [18–21] as well as a combination of both data classes [22]. Until recently, the molecular data sets suffered partly from incomplete coverage of families [18, 21], included only single or few (maximally six) loci [18–22] and led to partially discordant results with poorly supported basal nodes in the family level phylogenies obtained (see [12]). In the meantime, a comprehensive phylogenomic study has provided a well-supported phylogenetic backbone [12] that reconfirms inter-familial homoplasy in external morphological traits, as previously identified in morphology-based analyses [17, 22]. In contrast to external morphology, however, the potential of internal anatomy to support phylogenetic relationships within the pycnogonid crown group remains largely unexplored, as—with few exceptions (e.g., [23])—studies on internal organ systems mostly concentrate on single or few species [24–31].

Comparative neuroanatomical and neurodevelopmental studies across Arthropoda have elucidated evolutionary transformations of the central nervous system (CNS) during arthropod diversification [32, 33]. Repeatedly, this has yielded compelling morphological arguments weighing in on phylogenetic debates and increasing support for contested relationships [34–42]. Compared to many other arthropod lineages, however, the CNS of pycnogonids is poorly studied. Classical histological studies focusing on its gross structure were authored in various languages and are therefore frequently overlooked or only incompletely considered in recent works [26, 43–49]. These studies revealed anatomically separate segmental ganglia as a hallmark of the pycnogonid ventral nerve cord (VNC), in contrast to the fused prosomal neuromeres of euchelicerates [50–53]. Given the putative presence of separated ganglia in the VNC of some chelicerate/arthropod stem lineage representatives [54–56] as well as in extant mandibulates and arthropod outgroups (e.g., [57, 58]), this is likely to reflect the ancestral state of the chelicerate lineage [59]. Beyond gross anatomy, a handful of recent studies provided first insight into aspects of ventral ganglion architecture [10, 60–62]. But in keeping with the earlier accounts, only a subset of families was treated, with a predominance of easily obtainable groups, such as Ammotheidae, Callipallenidae, Pycnogonidae and Phoxichilidiidae. Accordingly, a comprehensive overview of the range of structural CNS variations in the pycnogonid crown group is currently impeded by limited taxon coverage.

Another characteristic feature of pycnogonid anatomy are prominent midgut diverticula that branch from the antero-posteriorly extending central midgut tube and project into the cheliphores (if present in adults) and all legs, and in some taxa also into the proboscis [3, 31]. Notably, rhythmical peristaltic contractions of the pycnogonid midgut and its diverticula have been noticed early on [43, 63]. This gut peristalsis has been recently shown to act as an important driver of hemolymph circulation and oxygen transport [64], which supports the comparatively weakly developed heart located dorsal to the central midgut tube [63, 65–67]. In most species hitherto studied, the diverticula represent simple linear extensions of the central midgut tube. However, more extensive, multi-branching diverticula have been reported for *Pycnogonum litorale* (Strøm, 1762), a common shallow water representative of the Pycnogonidae [44, 68, 69]. At this point, it remains unknown whether similar branching patterns occur in any other pycnogonid lineage, and a systematic comparison across all extant families is lacking.

Addressing the persistent gaps of knowledge pertaining to CNS and midgut structure in the pycnogonid crown group, this study pioneers the use of non-invasive micro-computed X-ray tomography (µCT) coupled to 3D reconstruction to obtain directly comparable in-situ representations of both organ systems across all nominal families. Beyond this, immunolabeling for cytoskeletal elements (two tubulin variants) and a marker for synaptic neuropil (synapsin) was conducted on dissected CNS whole mounts to gain more insight into the degree of fusion of ventral ganglia as well as the presence/absence of smaller nerves. Further, different genera of morphologically diverse families were included, to assess the extent of intra-familial pattern conservation versus variability. The results are mapped on the most comprehensive and best supported pycnogonid phylogeny available [12] to evaluate which of the CNS and midgut patterns studied (1) may be securely traced to the last common ancestor of the pycnogonid crown group and (2) may be phylogenetically informative with regard to the morphologically hitherto poorly supported inter-familial relationships.

## Results

### Data basis and order of descriptions

The descriptions are ordered according to families. For each nominal family, the structure of the CNS and the layout of the midgut—including the distal extensions of its leg diverticula—were in a first step assessed in overview µCT scans of at least one, sometimes also two or three species (Additional file 1: Table S1). Image stacks of higher magnification µCT scans of (part of) the trunk were subsequently used for the reconstruction of both organ systems. Results from tubulin and synapsin immunolabeling of CNS whole mounts complement the µCT descriptions where helpful. If additional data for more family members were generated, they are mentioned at the end of each family section. For practical (not phylogenetic) reasons, the family Nymphonidae is described first, as many adults of this lineage possess a fully segmented trunk with intersegmental longitudinal musculature as well as the complete set of functional cephalic appendages (in both sexes), which correlates with the presence of well-developed nerves innervating these structures. The descriptions of the other families focus predominantly on deviations from the nymphonid pattern. A full list of abbreviations used in the descriptions and figures is provided in Table 1.

**Table 1:** List of abbreviations used in the study

A–P	Anterior–posterior	LN	Leg nerve
AT	Anal tubercle	LP	Lateral process
BR	Brain	MG	Midgut
CEC	Circumesophageal connective	MIP	Maximum intensity projection
CHD	Cheliphore diverticulum	ON	Optic nerve
CHN	Cheliphore nerve	OT	Ocular tubercle
CLSM	Confocal laser scan microscopy	OVN	Ovigeral nerve
CNS	Central nervous system	PAN	Palpal nerve
CON	Connective	PBS	Phosphate-buffered saline
D	Diverticulum	PD	Proboscis diverticulum
DLM	Dorsal longitudinal musculature	RT	Room temperature
DPN	Dorsal proboscis nerve	SEG	Subesophageal ganglion
EY	Eye	TS	Trunk segment
GO	Gonads	VLM	Ventral longitudinal musculature
H	Heart	VNC	Ventral nerve cord
ISN	Intersegmental nerve	VPN	Ventral proboscis nerve
LD	Leg diverticulum	µCT	Micro-computed X-ray tomography
LG	Leg ganglion		

### Nymphonidae

#### ***Nymphon gracile*** Leach, 1814; Fig. 1

**Fig. 1:**
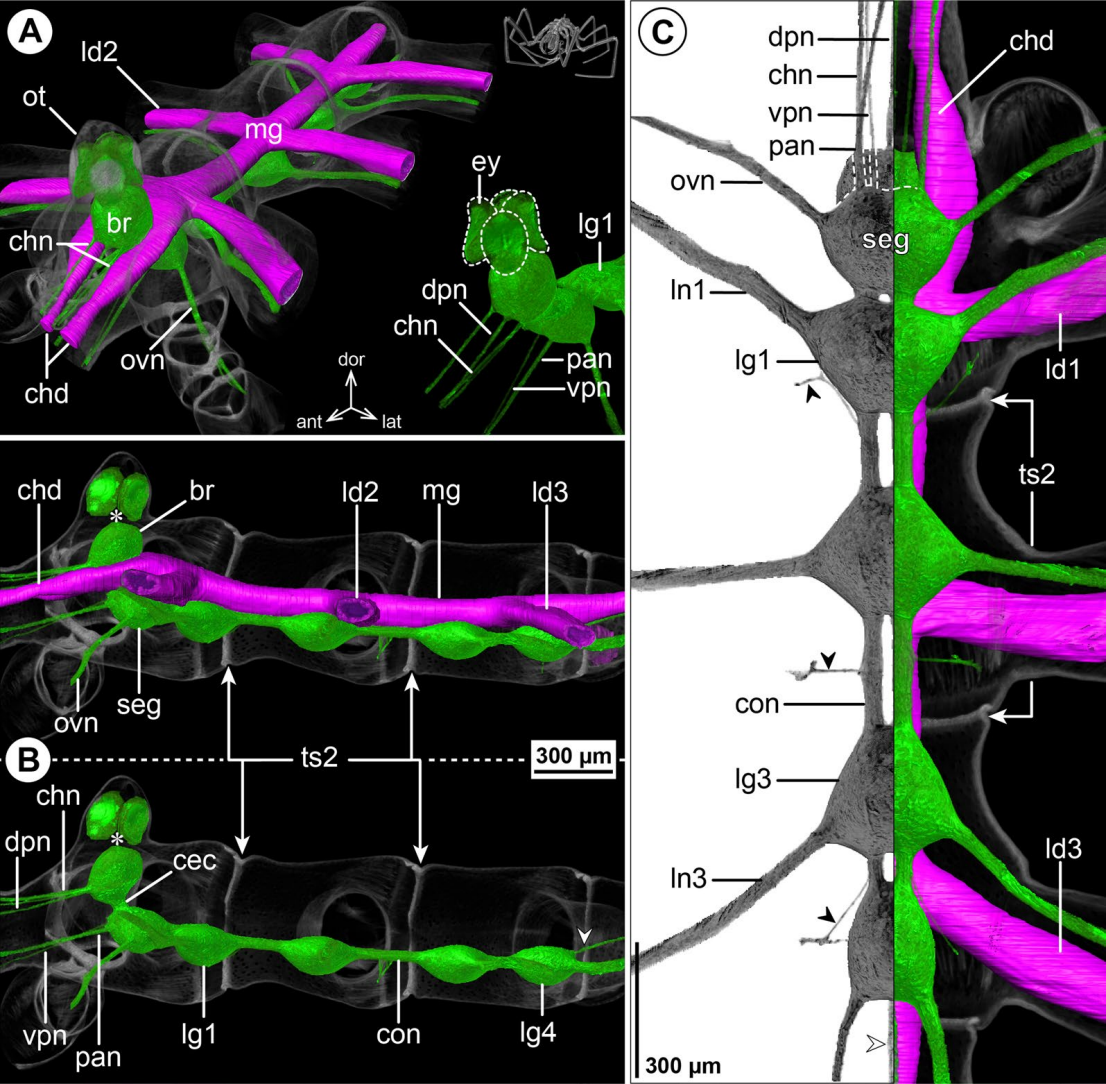
The central nervous system and midgut in the trunk of *Nymphon gracile* (Nymphonidae). Reconstructions of the CNS (3D volume rendering, green) and midgut (3D surface, magenta) based on a µCT scan of an adult male. The white arrowheads point to the origin of the posteriorly projecting proctodeal nerve. Asterisks indicate the incompletely reconstructed optic nerve (insufficient resolution). For better pattern visualization, all major nerves of the right body half were virtually removed in **A** (right side) and **B**. **A** Oblique antero-lateral view. Right top corner: overview of the complete specimen. Left side: complete CNS and midgut reconstruction. Right bottom corner: anterior portion of the CNS and eyes. **B** Lateral view of the CNS with and without the midgut structures (top and bottom, respectively). For reference, unreconstructed parts of the right body half are shown in transparent gray. **C** Ventral view. Left side: CNS in grayscale. The black arrowheads point to the intersegmental nerves. Right side: CNS and midgut. For reference, unreconstructed dorsal parts of the trunk are shown in transparent gray

**Fig. 2:**
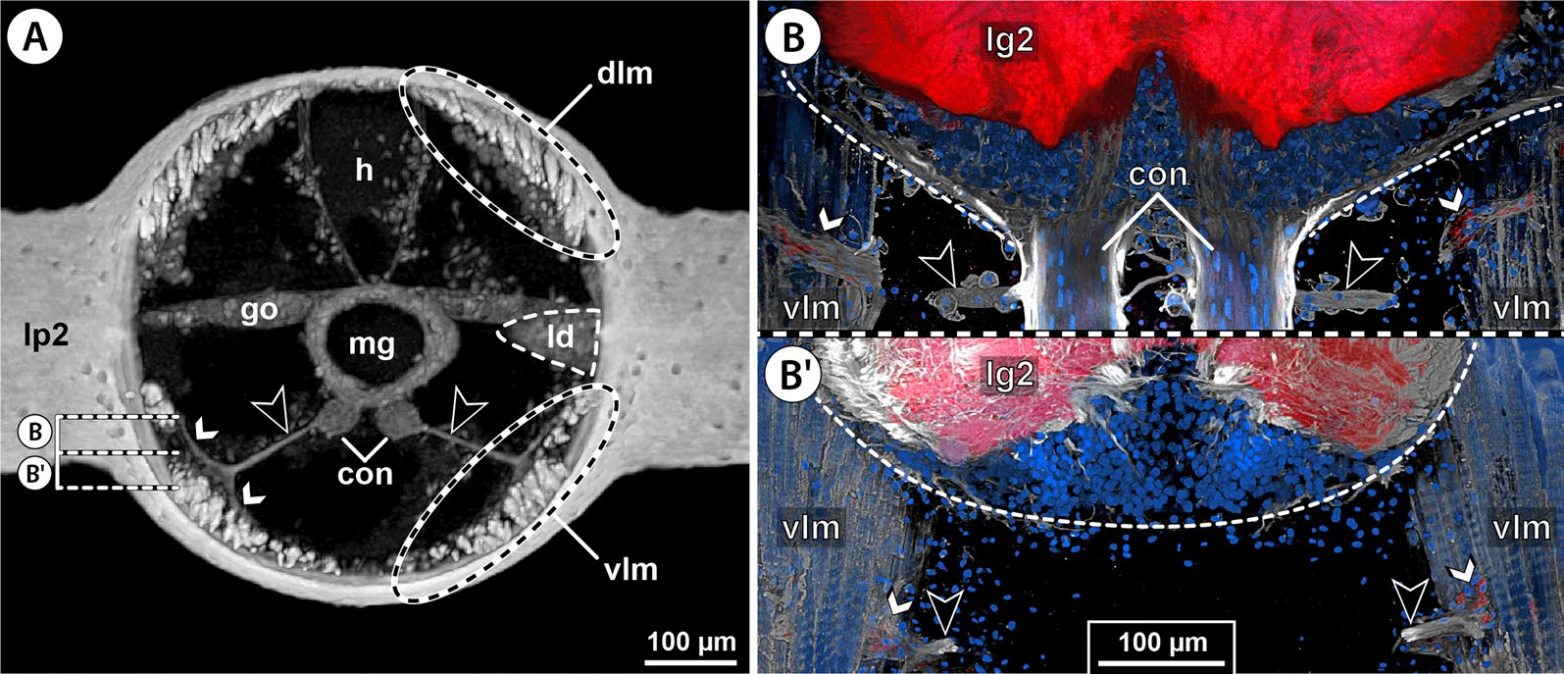
Intersegmental nerves in the ventral nerve cord of Nymphonidae. **A** *Nymphon gracile*, male, cross section through the posterior portion of trunk segment 2, µCT scan. The large black arrowheads point to the paired ISN. White arrowheads mark areas in which intersegmental nerve projections contact the ventral longitudinal musculature. The stippled brackets indicate the positions of the sections shown in **B** and **B’**. **B**, **B’** *N. grossipes*, male, CLSM scans (blend mode) of synapsin (red) and tyrosinated alpha-tubulin (gray) immunolabeling with nuclear counterstain (blue), horizontal sections, **B** lies dorsal to **B’**. The large black arrowheads indicate the paired intersegmental nerve. Note synapsin labeling at the neuromuscular junction (white arrowheads) of the ventral longitudinal musculature

The ovoid-shaped brain of *N. gracile* is located ventral to the ocular tubercle that bears two pairs of prominent eyes (Fig. 1A, B). Apart from the optic nerves (not labeled in µCT reconstructions; Fig. 1B), the unpaired dorsal proboscis nerve (DPN) projects from the antero-ventral brain surface and extends anteriorly through the cephalosoma’s narrow neck toward the dorsal proboscis antimere (Fig. 1). The paired cheliphoral nerve (CHN) emerges lateral to the DPN, bifurcates at a short distance from the brain and proceeds next to the cheliphoral gut diverticulum (CHD) through the anterior neck (Fig. 1A, B). A short soma-free circumesophageal connective (CEC) spans from the brain to the compact subesophageal ganglion (SEG), which lies slightly posterior to the ovigeral lateral process (Fig. 1B, C). The SEG is comprised of the fused palpal and ovigeral neuromeres. Three paired nerves project from it: (1) the relatively delicate ventral proboscis nerve (VPN) emerges at its antero-medial surface and extends anteriorly into the ipsilateral ventral proboscis antimere, (2) the palpal nerve (PAN) has its root lateral to the VPN and serves the palp, and (3) the ovigeral nerve (OVN) projects antero-ventrally from the SEG’s lateral side into the oviger (Fig. 1; Additional file 2: Fig. S1A). In adults, the SEG and the ganglion of the first leg pair (LG1) almost touch, being separated by a very short connective only (Fig. 1C). In subadult specimens, both ganglia are touching, but their soma cortices and neuropil cores are anatomically separate (Additional file 2: Fig. S1A). The LG1 is completely included in the cephalosoma and its segmental nerve extends laterally into the first leg. Posterior to LG1, the connectives between the following ventral ganglia (LG2-4) are more elongated (Fig. 1B, C). Delicate intersegmental nerves (ISNs) project ventro-laterally from the connectives between LG2 to LG4 (Fig. 1C) and contact the ventral longitudinal musculature of the free trunk segments (Fig. 2). While LG2 is located centrally in its trunk segment, LG3 is positioned in the anterior half of trunk segment 3, which additionally includes LG4 in its posterior half (Fig. 1B, C). As a consequence of this anterior shift, trunk segment 4 does not contain a ganglion and the segmental nerves of LG3 and LG4 project postero-laterally and posteriorly, respectively, into the lateral processes bearing the legs (Fig. 1C). None of the segmental nerves in the VNC bifurcate prior to entering their respective lateral process. (Fig. 1C). At the dorso-posterior side of LG4, a paired proctodeal nerve extends from a small median protrusion (= remnant of transient posterior ganglion anlagen) along the midline, and runs directly ventral to the central midgut toward the anal tubercle (Fig. 1B, C).

The layout of the midgut is simple. The central gut tube runs dorsal to the VNC, extending from the posterior brain surface toward the anal tubercle (Figs. 1A, B, 2A). At the tube’s antero-lateral margin, the paired cheliphore diverticulum (CHD) emerges, embraces the CEC laterally and projects anteriorly into the cheliphore scape (Fig. 1A, C). Additionally, a linear leg diverticulum (LD) projects into each leg, displaying a well-developed lumen up to the distal end of tibia 2, before continuing as a compact cell strand to the tarsus-propodus border. Each LD branches off the central gut tube in its respective trunk segment, LD1 and LD2 emerging next to their lateral process, LD3 and LD4 only slightly anterior to it (Fig. 1A, C; LD4 not shown).

#### ***Pentanymphon antarcticum*** Hodgson, 1904; Additional file 3: Figure S2

From the brain to LG 3, the layout of the CNS in the decapodous *P. antarcticum* is virtually identical to the octopodous *N. gracile* (Additional file 3: Fig. S2A-C). Posterior to LG3, LG4 is located at the border of trunk segments 3 and 4, whereas LG5 is positioned in the anterior half of trunk segment 4 (Additional file 3: Fig. S2B, C). Accordingly, the ultimate LG5 in *P. antarcticum* is shifted further anteriorly with respect to its trunk segment than the ultimate LG4 in *N. gracile* (compare Fig. 1C and Additional file 3: Fig. S2C).

As in *N. gracile*, the divergence points of the leg diverticula from the central midgut are not forward-shifted but lie close to the lateral process of the respective trunk segments (Additional file 3: Fig. S2A–C).

#### Additional nymphonid representatives studied

Tubulin and synapsin immunolabeling of CNS dissections of the two octopodous species *N. grossipes* (Fabricius, 1780) and *N. macronyx* Sars, 1877 show a corresponding number of ventral ganglia with their suites of emanating nerves, as well as similar relative proportions of the connectives as *N. gracile*.

### Callipallenidae

#### ***Parapallene avida*** Stock, 1973; Fig. 3

**Fig. 3:**
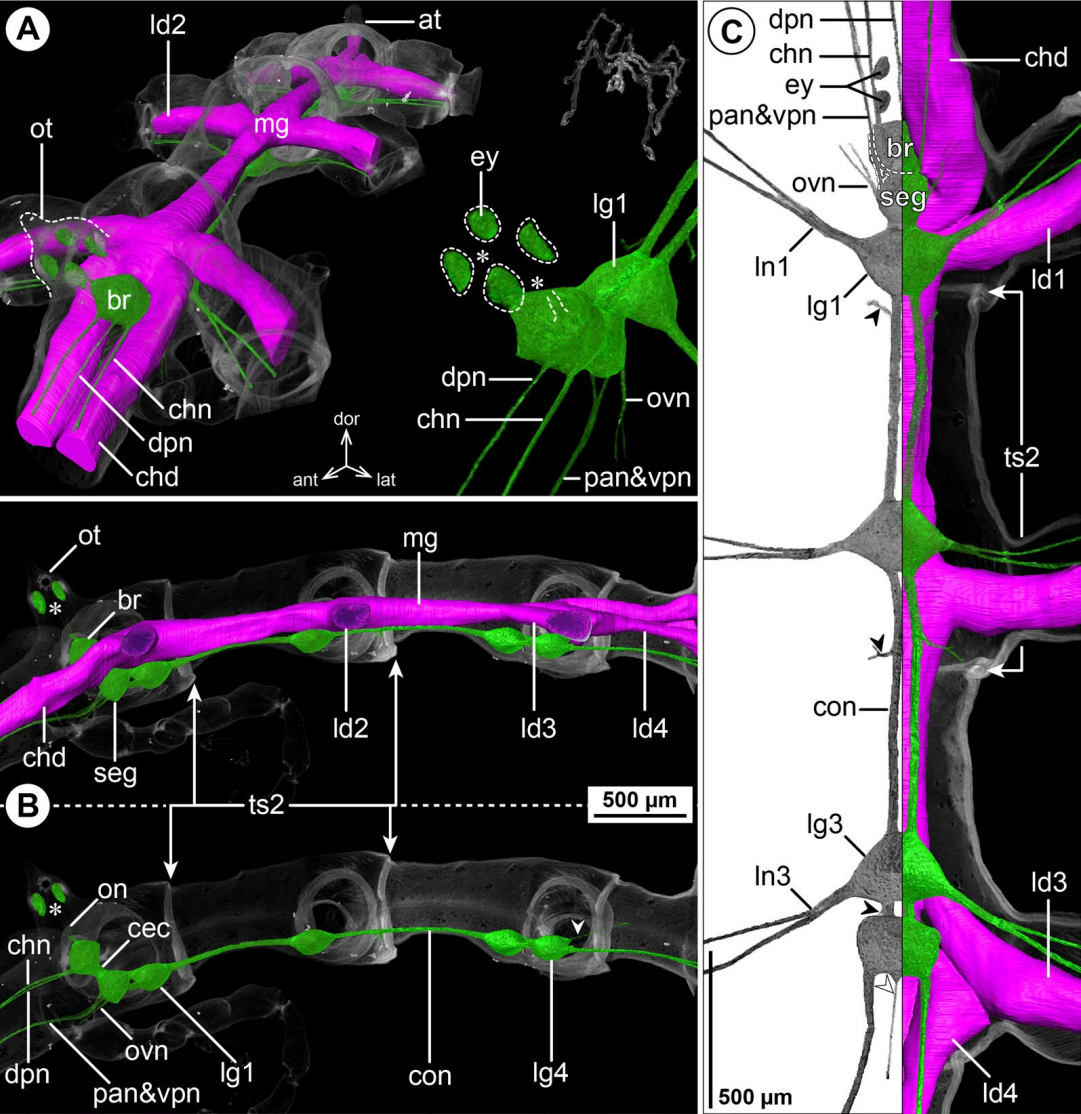
The central nervous system and midgut in the trunk of *Parapallene avida* (Callipallenidae). Reconstructions of the CNS (3D volume rendering, green) and midgut (3D surface, magenta) based on a µCT scan of an adult female. The white arrowheads point to the origin of the posteriorly projecting proctodeal nerve. Asterisks indicate the incompletely reconstructed optic nerve (insufficient resolution). For better pattern visualization, all major nerves of the right body half were virtually removed in **A** (right side) and **B**. **A** Oblique antero-lateral view. Right top corner: overview of the complete specimen. Left side: complete CNS and midgut reconstruction. Right bottom corner: anterior portion of the CNS and eyes. **B** Lateral view of the CNS with and without the midgut structures (top and bottom, respectively). For reference, unreconstructed parts of the right body half are shown in transparent gray. **C** Ventral view. Left side: CNS in grayscale. The black arrowheads point to the intersegmental nerves. Right side: CNS and midgut. For reference, unreconstructed dorsal parts of the trunk are shown in transparent gray

The callipallenid *Pa. avida* is characterized by a very slender habitus with elongated, tube-like trunk segments. The relative position of the brain to the ocular tubercle, the number of ventral ganglia and their positions relative to the segment borders concurs with the pattern of octopodous nymphonids (Fig. 3B, C). The connectives between LG1-3 are extremely elongated, whereas the connective between LG3 and LG4 is very short, owing to the anterior shift of LG4 into trunk segment 3 (Fig. 3B, C). In spite of the absence of palps (in both sexes), a nerve emerges in a corresponding position from the anterior side of the SEG, lateral to the VPN. At a short distance from the SEG, both nerves converge and project close to each other through the neck (Fig. 3A, C). The remaining segmental nerves in the VNC split into two main branches before entering their respective lateral processes (Fig. 3C).

The midgut layout is likewise similar to octopodous nymphonids, featuring leg diverticula that extend all the way into the propodus. Within the trunk, LD4 branches off the central tube already in segment 3, directly posterior to LD3 (Fig. 3B, C). Thus, it mirrors the forward shift of LG4, which lies ventral to its divergence point.

#### ***Callipallene tiberii*** (Dohrn, 1881); Fig. 4

**Fig. 4:**
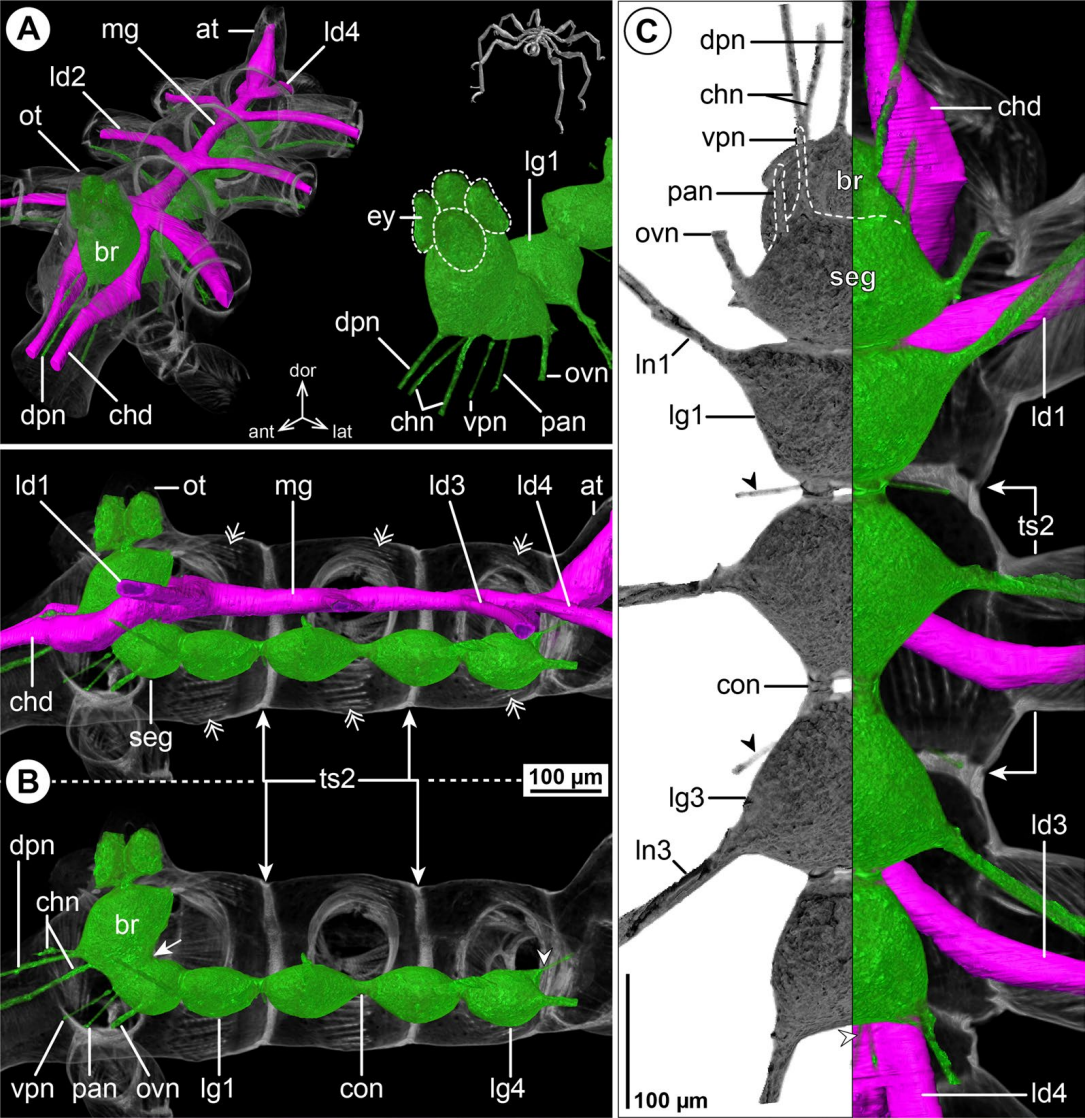
The central nervous system and midgut in the trunk of *Callipallene tiberii* (Callipallenidae). Reconstructions of the CNS (3D volume rendering, green) and midgut (3D surface, magenta) based on a µCT scan of an adult female. The white arrowheads point to the origin of the posteriorly projecting proctodeal nerve. For better pattern visualization, all major nerves of the right body half were virtually removed in **A** (right side) and **B**. **A** Oblique antero-lateral view. Right top corner: overview of the complete specimen. Left side: complete CNS and midgut reconstruction. Right bottom corner: anterior portion of the CNS and eyes. **B** Lateral view of the CNS with and without the midgut structures (top and bottom, respectively). For reference, unreconstructed parts of the right body half are shown in transparent gray. The double arrows point to intersegmental longitudinal musculature. The white arrow highlights the fusion area of brain and subesophageal ganglion. **C** Ventral view. Left side: CNS in grayscale. The black arrowheads point to the intersegmental nerves. Right side: CNS and midgut. For reference, unreconstructed dorsal parts of the trunk are shown in transparent gray

*Callipallene tiberii* is more compact than *Pa. avida*, although the lateral processes are still distinctly set apart from each other (Fig. 4A, C). Also the CNS is more compact, featuring only very short connectives between the ventral ganglia (Fig. 4B, C). Notably, a soma-free CEC is not present, i.e., the brain and SEG form a compact mass that surrounds the esophagus (Fig. 4B). In the VNC, LG3 is shifted halfway into trunk segment 2 and LG4 is completely contained in trunk segment 3 (Fig. 4B, C). The CHN is the only segmental nerve showing a distinct bifurcation prior to entering its appendage; it emerges already as two separate branches from the brain’s soma cortex (Fig. 4A, B). While ISNs are present between LG1 to LG3, a corresponding nerve could not be identified between LG3 and LG4 in the µCT data (Fig. 4C). This apparent lack of the posterior-most ISN coincides with a less distinct segment border and less developed intersegmental longitudinal musculature between trunk segments 3 and 4 (Fig. 4B). However, tubulin immunolabeling in the similar congener *Callipallene brevirostris* (Johnston, 1837) reveals a very delicate ISN in this position (Additional file 4: Fig. S3A), which may point to a lack of resolution in the *C. tiberii* µCT scans .

The midgut layout shows no remarkable deviations from the nymphonid pattern. Each LD extends with a distinct lumen up to the tibia 2-tarsus border of its respective leg. In contrast to the callipallenid *Pa. avida*, the LD4 diverges not within trunk segment 3, but at the border of trunk segments 3 and 4 (Fig. 4B, C).

#### Other callipallenid representatives studied

The CNS and midgut layout in *Callipallene brevirostris* (Johnston, 1837) closely resembles its congener of *C. tiberii* (Additional file 5: Fig. S4A). Also the robust *Stylopallene cheilorhynchus* Clark, 1963 exhibits very similar patterns (Additional file 6: Fig. S5), including the absence of a soma-free CEC (Additional file 6: Fig. S5C). Like *C. tiberii*, *S. cheilorhynchus* shows an indistinct separation of trunk segments 3 and 4. In *S. cheilorhynchus*, this is accompanied by the lack of longitudinal musculature between both segments (Additional file 6: Fig. S5C), and an ISN between LG3 and LG4 could neither be identified in the µCT scan nor in tubulin-immunolabeled samples (Additional files 4 and 6: Figs. S3B; S5B). Notably, while an ISN between LG2 and LG3 could also not be confidently traced in the µCT data (Additional file 6: Fig. S5B), tubulin labeling reveals its origin at the connective, from where it extends through the anterior portion of the LG3 soma cortex and emerges at its antero-lateral margin embedded in a strand of connective tissue (Additional file 4: Fig. S3B). In correspondence to *Pa. avida*, the lumen of the leg diverticula extends all the way into the propodus of the legs.

### Ascorhynchidae

#### ***Ascorhynchus ramipes*** (Böhm, 1879); Fig. 5

**Fig. 5:**
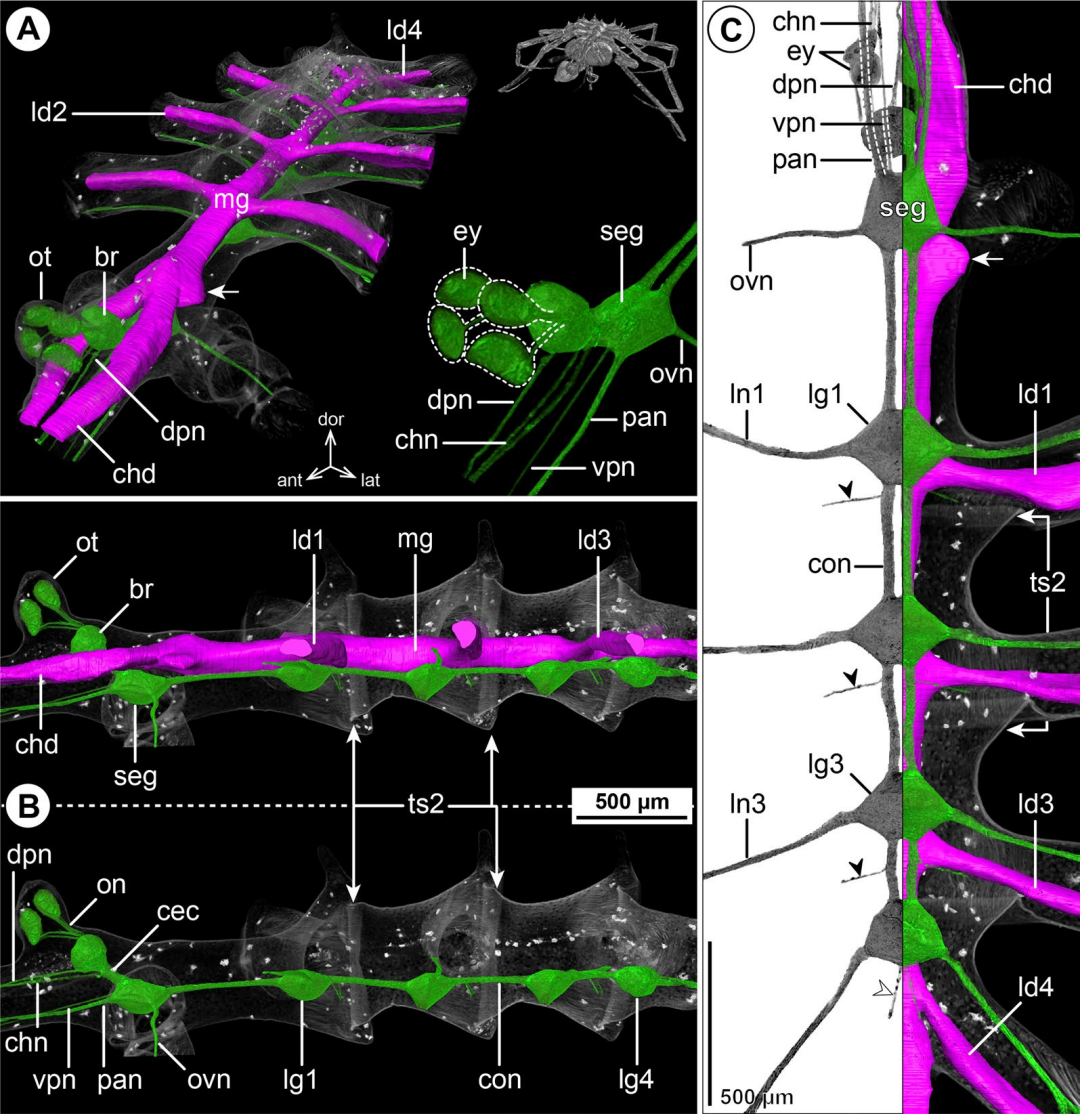
The central nervous system and midgut in the trunk of *Ascorhynchus ramipes* (Ascorhynchidae). Reconstructions of the CNS (3D volume rendering, green) and midgut (3D surface, magenta) based on a µCT scan of an adult male. The white arrowheads point to the origin of the posteriorly projecting proctodeal nerve. The white arrows indicate a small lateral midgut protrusion near the subesophageal ganglion. For better pattern visualization, all major nerves of the right body half were virtually removed in **A** (right side) and **B**. **A** Oblique antero-lateral view. Right top corner: overview of the complete specimen bearing egg packages on the ovigers. Left side: complete CNS and midgut reconstruction. Right bottom corner: anterior portion of the CNS and eyes. **B** Lateral view of the CNS with and without the midgut structures (top and bottom, respectively). For reference, unreconstructed parts of the right body half are shown in transparent gray. **C** Ventral view. Left side: CNS in grayscale. The black arrowheads point to the intersegmental nerves. Right side: CNS and midgut. For reference, unreconstructed dorsal parts of the trunk are shown in transparent gray

In this slender species, the cephalosoma is markedly elongated, featuring a distinct separation of the lateral processes of the oviger and leg 1 (Fig. 5A, C). The brain lies slightly posterior to the ocular tubercle, which is located even more anterior than the ovigeral lateral process (Fig. 5A, B). Conspicuously, the SEG is positioned as far anterior as the ovigeral lateral process and the connective to LG1 is the longest of the VNC (Fig. 5B, C). The structure of the more posterior VNC shows no remarkable features, apart from a more elongated connective between LG3 and LG4 compared to, e.g., the equally slender callipallenid *Pa. avida* (Figs. 5C, 3C). With the exception of the CHN, which projects as two separate branches from the brain (Fig. 5A), none of the segmental nerves bifurcate prior to entering its lateral process (Fig. 5C). In line with the well-developed palps and ovigers, the PAN and OVN are prominent and almost equal the leg nerves in diameter (Fig. 5C).

The LDs diverge from the midgut tube within their respective trunk segments, in close vicinity to the lateral processes they target (Fig. 5C). The LD lumen extends to the distal end of tibia 1, from whereon the leg articles are extremely slender and without recognizable LD. Additionally, a small lateral midgut protrusion is found near the ovigeral lateral process, but does not extend into it (Fig. 5A, C).

#### Additional ascorhynchid representatives studied

Micro-CT analysis of *Ascorhynchus castellioides* Stock, 1957 with its slightly more compact habitus than the slender *As. ramipes* shows nonetheless a distinct spatial separation of brain and SEG from LG1 (Additional file 5: Fig. S4B) and a corresponding central midgut branching pattern (the extension of the LD lumen into the legs was not traced).

Further, similar proportions of the connectives were confirmed in CNS whole mounts of *As. glaberrimus* Schimkewitsch, 1913 and *As. auchenicus* (Slater, 1879) (Additional file 2: Fig. S1H). Also *Nymphonella tapetis* Ohshima, 1927, a species traditionally considered as part of the Ascorhynchidae, displays clearly separated ganglia, including the long connective between SEG and LG1 (Additional file 2: Fig. S1I).

### Endeidae

#### ***Endeis spinosa*** (Montagu, 1808); Fig. 6

**Fig. 6:**
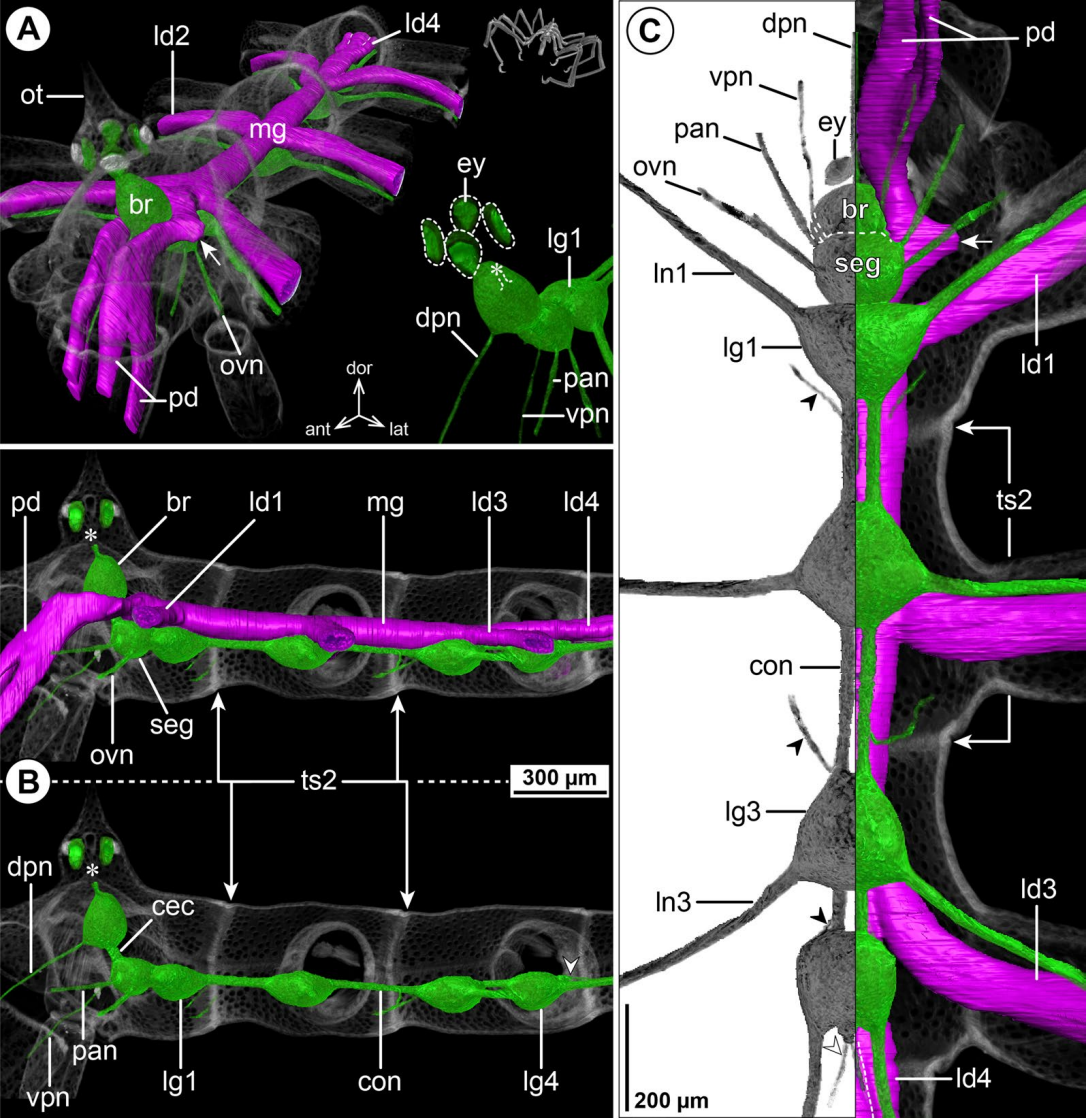
The central nervous system and midgut in the trunk of *Endeis spinosa* (Endeidae). Reconstructions of the CNS (3D volume rendering, green) and midgut (3D surface, magenta) based on a µCT scan of an adult male. The white arrowheads point to the origin of the posteriorly projecting proctodeal nerve. The white arrows highlight a small lateral midgut protrusion near the subesophageal ganglion. Asterisks indicate the incompletely reconstructed optic nerve (insufficient resolution). For better pattern visualization, all major nerves of the right body half were virtually removed in **A** (right side) and **B**. **A** Oblique antero-lateral view. Right top corner: overview of the complete specimen. Left side: complete CNS and midgut reconstruction. Right bottom corner: anterior portion of the CNS and eyes. **B** Lateral view of the CNS with and without the midgut structures (top and bottom, respectively). For reference, unreconstructed parts of the right body half are shown in transparent gray. **C** Ventral view. Left side: CNS in grayscale. The black arrowheads point to the intersegmental nerves. Right side: CNS and midgut. For reference, unreconstructed dorsal parts of the trunk are shown in transparent gray

The overall CNS structure and proportions are very similar to the octopodous *N. gracile* (Fig. 6B, C). Concurrent with the lack of cheliphores in adult *E. spinosa* (in both sexes), the CHN could not be traced in the µCT scan (Fig. 6A, B). Despite the absence of palps, the PAN is clearly discernible (Fig. 6B, C). It projects to extrinsic ventral proboscis musculature and seems to extend even further into the ipsilateral ventral proboscis antimere. In males, the OVN is more prominent than in the oviger-lacking females (not shown). Since the cephalosoma lacks a distinct, anteriorly directed neck, the unpaired DPN and paired VPN project immediately into the antero-ventrally directed proboscis (Fig. 6A, B).

The midgut diverticula show a few deviations from the nymphonid layout. In line with the absent cheliphore, a CHD is missing. Instead, the diverticulum that circles the CEC projects antero-ventrally into the proboscis, where it bifurcates into a dorsal and a ventral branch (Fig. 6A, B). These branches flank the upward directed arm of the Y-shaped pharynx for about two thirds of the proboscis length. At the level of the esophagus, the midgut features a laterally protruding bulge, similar to the one in the ascorhynchid *As. ramipes* (Fig. 6A, C). The divergence points of LD1 to LD3 lie within the respective trunk segments, with LD3 being slightly forward-shifted in relation to the lateral process it targets (Fig. 6C). By contrast, LD4 emerges already in the posterior half of trunk segment 3 and runs in parallel to the central midgut tube (Fig. 6B, C) prior to extending into the lateral process of trunk segment 4. The lumen of each LD extends all the way into the propodus.

### Phoxichilidiidae

#### ***Phoxichilidium femoratum*** (Rathke, 1799); Fig. 7

**Fig. 7:**
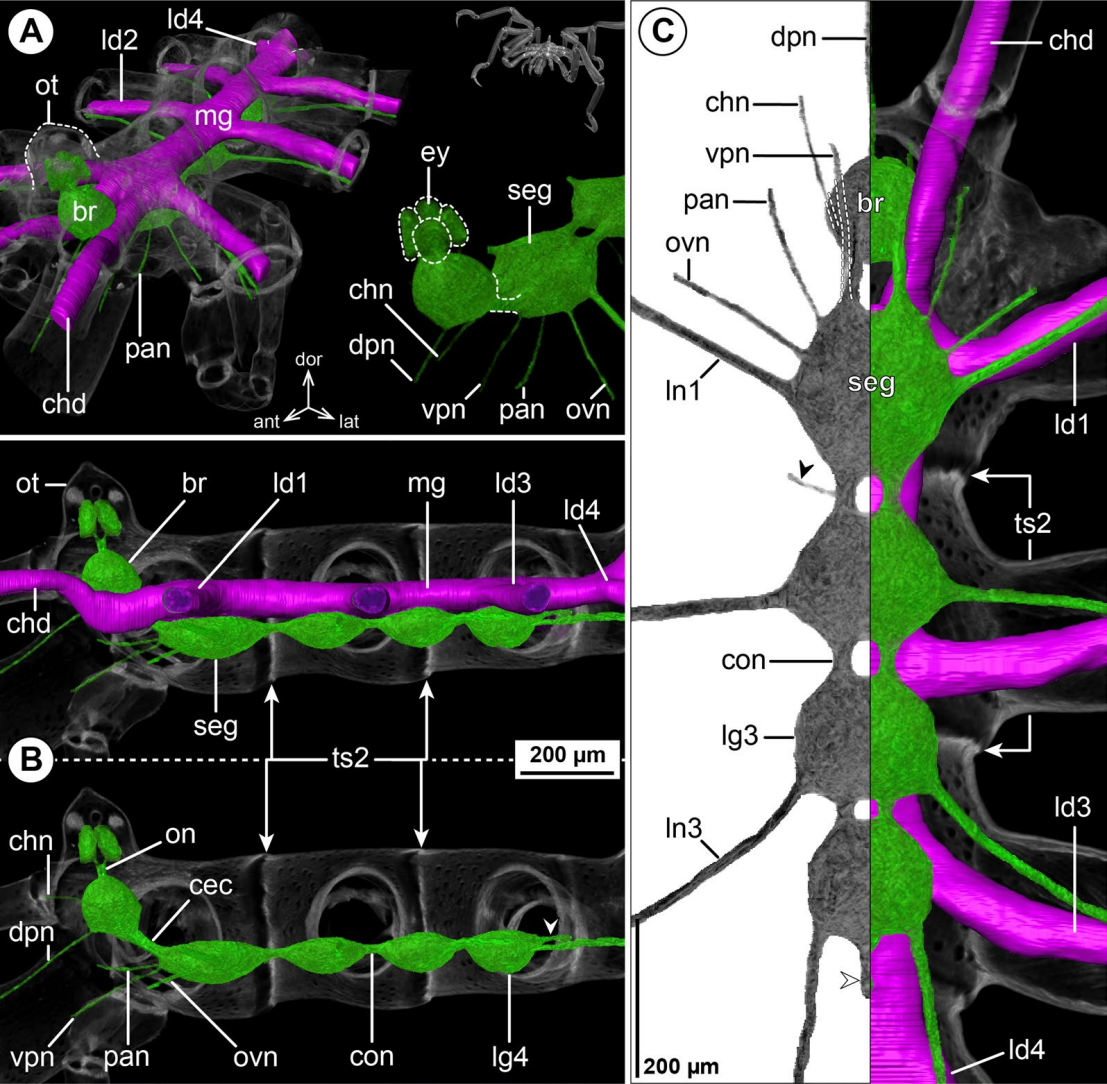
The central nervous system and midgut in the trunk of *Phoxichilidium femoratum* (Phoxichilidiidae). Reconstructions of the CNS (3D volume rendering, green) and midgut (3D surface, magenta) based on a µCT scan of an adult male. The white arrowheads point to the origin of the posteriorly projecting proctodeal nerve. For better pattern visualization, all major nerves of the right body half were virtually removed in **A** (right side) and **B**. **A** Oblique antero-lateral view. Right top corner: overview of the complete specimen. Left side: complete CNS and midgut reconstruction. Right bottom corner: anterior portion of the CNS and eyes. **B** Lateral view of the CNS with and without the midgut structures (top and bottom, respectively). For reference, unreconstructed parts of the right body half are shown in transparent gray. **C** Ventral view. Left side: CNS in grayscale. The black arrowhead points to an intersegmental nerve. Right side: CNS and midgut. For reference, unreconstructed dorsal parts of the trunk are shown in transparent gray

Deviating from all families covered so far, the VNC of *Ph. femoratum* displays an extended SEG that represents a fusion product of the palpal, ovigeral and leg 1 neuromeres and features a contiguous soma cortex and neuropil core (Fig. 7B, C; Additional file 2: Fig. S1G). Despite the lack of palps in both sexes, a nerve corresponding to the PAN extends toward the lateral base of the proboscis (Fig. 7A, B; Additional file 2: Fig. S1G). While the OVN of males is slightly more prominent than the PAN, it is less developed in the oviger-less females (compare Fig. 7A, C and Additional file 2: Fig. S1G). All ventral ganglia are connected by short connectives. In the µCT scans, ISNs were difficult to identify (Fig. 7C), but tubulin labeling of CNS whole mounts confirms their presence (Additional file 4: Fig. S3C).

The midgut layout shows no remarkable features (Fig. 7A, C). The divergence point of LD4 from the gut tube is only slightly forward-shifted, lying near the border of trunk segments 3 and 4 (Fig. 7A, B). The lumen of each LD extends to the tibia 2-tarsus border.

#### Additional phoxichilidiid representatives studied

In the minute and more compact *Anoplodactylus pygmaeus* (Hodge, 1864), the VNC lacks elongated connectives. In correspondence to *Ph. femoratum*, the palpal, ovigeral and leg 1 neuromeres form an extended SEG, whereas the remaining leg ganglia are anatomically separate (Additional file 2: Fig. S1J). A corresponding CNS structure was confirmed for the larger *A. australis* (Hodgson, 1914).

### Pallenopsidae

#### ***Pallenopsis*** cf. ***aulaeturcarum*** Dömel & Melzer, 2019; Fig. 8

**Fig. 8:**
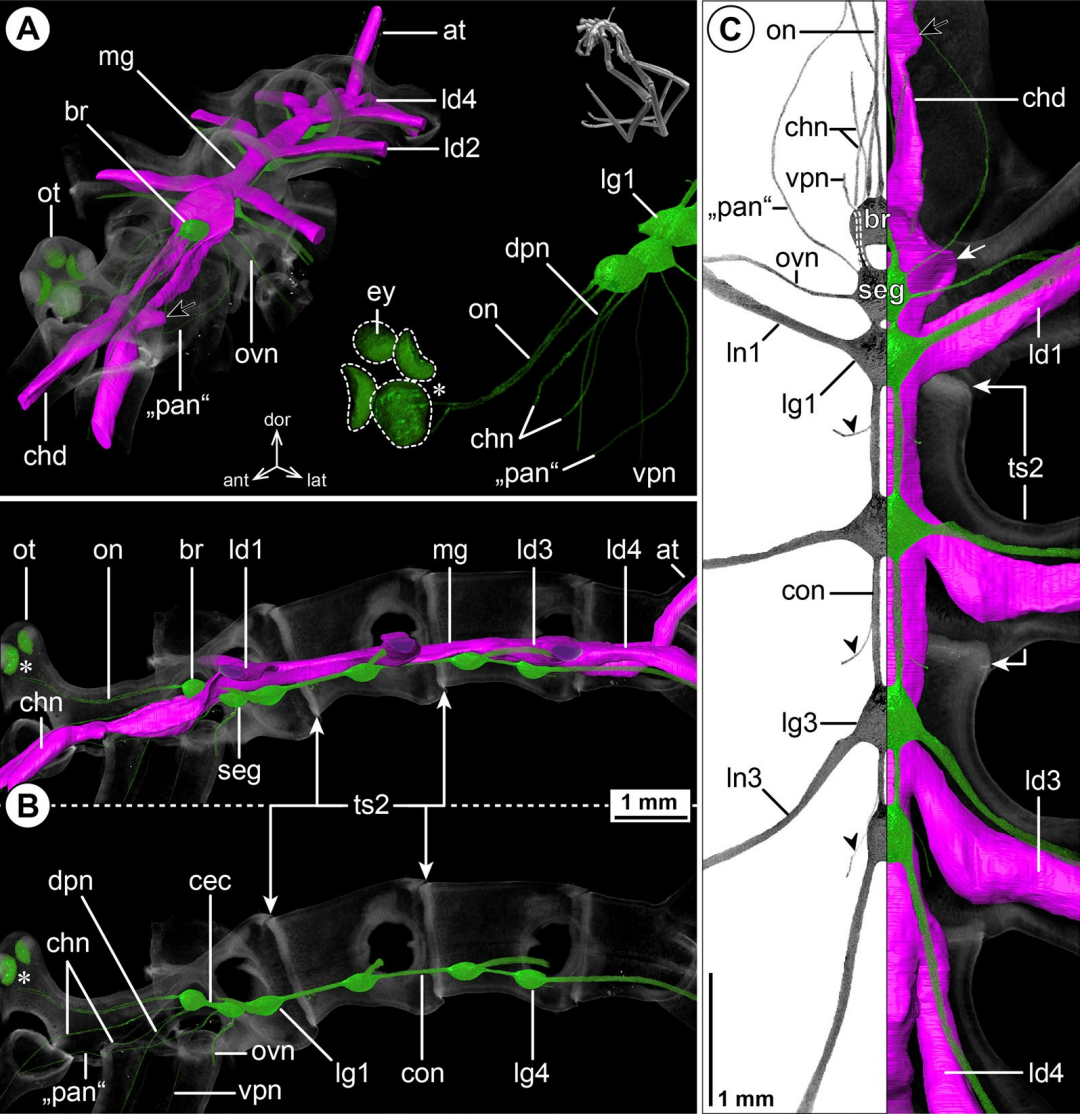
The central nervous system and midgut in the trunk of *Pallenopsis* cf. *aulaeturcarum* (Pallenopsidae). Reconstructions of the CNS (3D volume rendering, green) and midgut (3D surface, magenta) based on a µCT scan of an adult male. The black arrows point to a small lateral protrusion of the cheliphore diverticulum. Asterisks indicate the incompletely reconstructed optic nerve (insufficient resolution). For better pattern visualization, all major nerves of the right body half were virtually removed in **A** (right side) and **B**. The palpal nerve is labeled in quotation marks (“pan”) as it could not be traced into the reduced palp but projects to the cheliphore. **A** Oblique antero-lateral view. Right top corner: overview of the specimen. Left side: complete CNS and midgut reconstruction. Right bottom corner: anterior portion of the CNS and eyes. **B** Lateral view of the CNS with and without the midgut structures (top and bottom, respectively). For reference, unreconstructed parts of the right body half are shown in transparent gray. **C** Ventral view. Left side: CNS in grayscale. The black arrowheads point to the intersegmental nerves. Right side: CNS and midgut. The white arrow highlights a small lateral midgut protrusion near the subesophageal ganglion. For reference, unreconstructed dorsal parts of the trunk are shown in transparent gray

The position of the brain and ventral ganglia as well as the origin of the major nerves do not show any striking deviations from the nymphonid pattern (Fig. 8B, C). However, as the ocular tubercle of pallenopsids is positioned at the anterior margin of the cephalosoma, the paired optic nerve connecting the eyes and brain is much longer than in most other families (Fig. 8A, B). The anteriorly projecting CHN bifurcates at a short distance from the brain (Fig. 8A). None of the other segmental nerves show a major split prior to entering their lateral process/appendage (Fig. 8A, C). The PAN loops toward the base of the one-articled palp but could not be traced into the latter. Instead, it continues in an anterior direction to the base of the cheliphore (Fig. 8A, B).

The midgut layout displays only few deviations from the nymphonid pattern. While LD3 diverges slightly anterior to its lateral process in trunk segment 3, LD4 shows a more pronounced forward shift, emerging at the border of trunk segments 3 and 4 (Fig. 8B, C). Further, some of the LDs show a proximal widening prior to entering the lateral process (Fig. 8C). From thereon, the LD lumen extends to the tibia 2-tarsus border. At the anterior end, the midgut tube has a small lateral protrusion (Fig. 8C), comparable to the one observed in Ascorhynchidae and Endeidae. More anteriorly, the CHD exhibits a similar lateral protrusion, proximal to the insertion of the cheliphore scape (Fig. 8A, C).

#### Additional pallenopsid representatives studied

In correspondence to *P.* cf. *aulaeturcarum*, a similar long distance between ocular tubercle and brain was confirmed in a µCT overview scan of a subadult *P. vanhoeffeni* Hodgson, 1915 (Additional file 5: Fig. S4C). Additionally, an equivalent array of the brain and ventral ganglia and their nerve roots was found in CNS whole mounts *P.* cf. *obstaculumsuperavit* Dömel, 2019 and in subadults of another undetermined *Pallenopsis* sp.

### Ammotheidae

#### ***Achelia echinata*** Hodge, 1864; Fig. 9

**Fig. 9:**
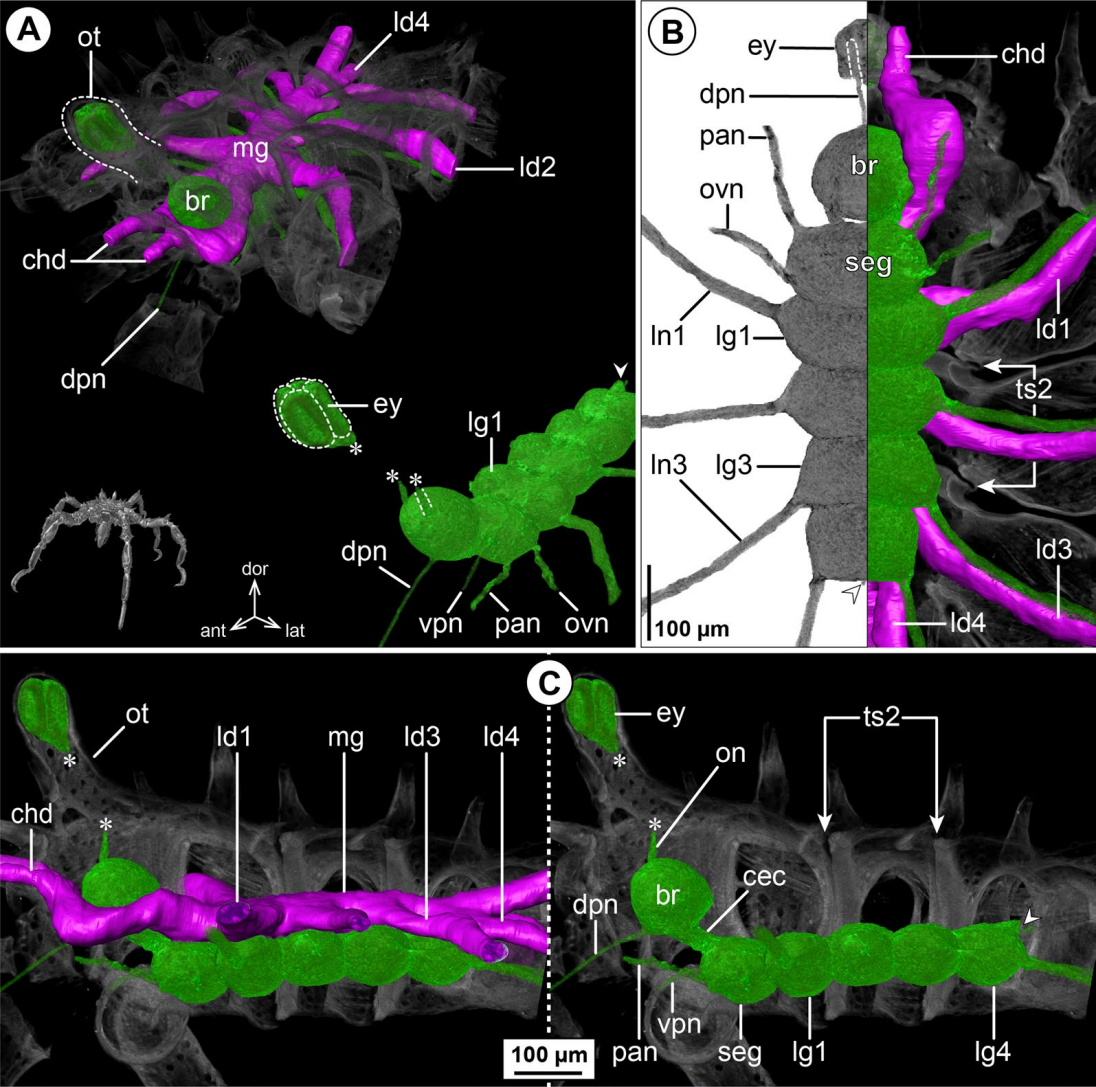
The central nervous system and midgut in the trunk of *Achelia echinata* (Ammotheidae). Reconstructions of the CNS (3D volume rendering, green) and midgut (3D surface, magenta) based on a µCT scan of an adult male. The white arrowheads point to the origin of the posteriorly projecting proctodeal nerve. Asterisks indicate the incompletely reconstructed optic nerve (insufficient resolution and tissue damage). For better pattern visualization, all major nerves of the right body half were virtually removed in **A** (right side) and **C**. **A** Oblique antero-lateral view. Left bottom corner: overview of the specimen. Top: complete CNS and midgut reconstruction. Right bottom corner: CNS and eyes. **B** Ventral view. Left side: CNS in grayscale. Right side: CNS and midgut. For reference, unreconstructed dorsal parts of the trunk are shown in transparent gray. **C** Lateral view of the CNS with and without the midgut structures (left and right, respectively). For reference, unreconstructed parts of the right body half are shown in transparent gray

In the compact *A. echinata*, the CEC is the only soma-free connective (Fig. 9C). All VNC ganglia touch each other and the ultimate LG4 is fully contained in trunk segment 3 (Fig. 9B, C). The ventral ganglia are anatomically distinct, with separate soma cortices and neuropil cores (Fig. 9B, C; Additional file 2: Fig. S1C). A CHN extending to the small cheliphores with atrophied chelae could not be identified in the µCT scans studied (Fig. 9A). Of all other cephalic main nerves (DPN, VPN, PAN, OVN), at least the proximal portions could be traced (Fig. 9A, C) and in the SEG, their presence was additionally confirmed by tubulin immunolabeling (Additional file 2: Fig. S1C). Between the LGs, ISNs could not be reliably reconstructed from the µCT data (Fig. 9A, B). By contrast, tubulin-labeled CNS whole mounts reveal delicate ISNs between LG1 to LG3, while the ISN between LG3 and LG4 is lacking (Additional file 4: Fig. S3D). The latter coincides with the external fusion of trunk segments 3 and 4 (Fig. 9C) and the absence of intersegmental longitudinal musculature.

The midgut LDs diverge from the central tube at the level of the corresponding LG, i.e., LD4 is forward-shifted and arises in the posterior half of trunk segment 3 (Fig. 9B). Each LD extends to the tibia 2-tarsus border.

#### ***Tanystylum orbiculare*** Wilson, 1878; Fig. 10

**Fig. 10:**
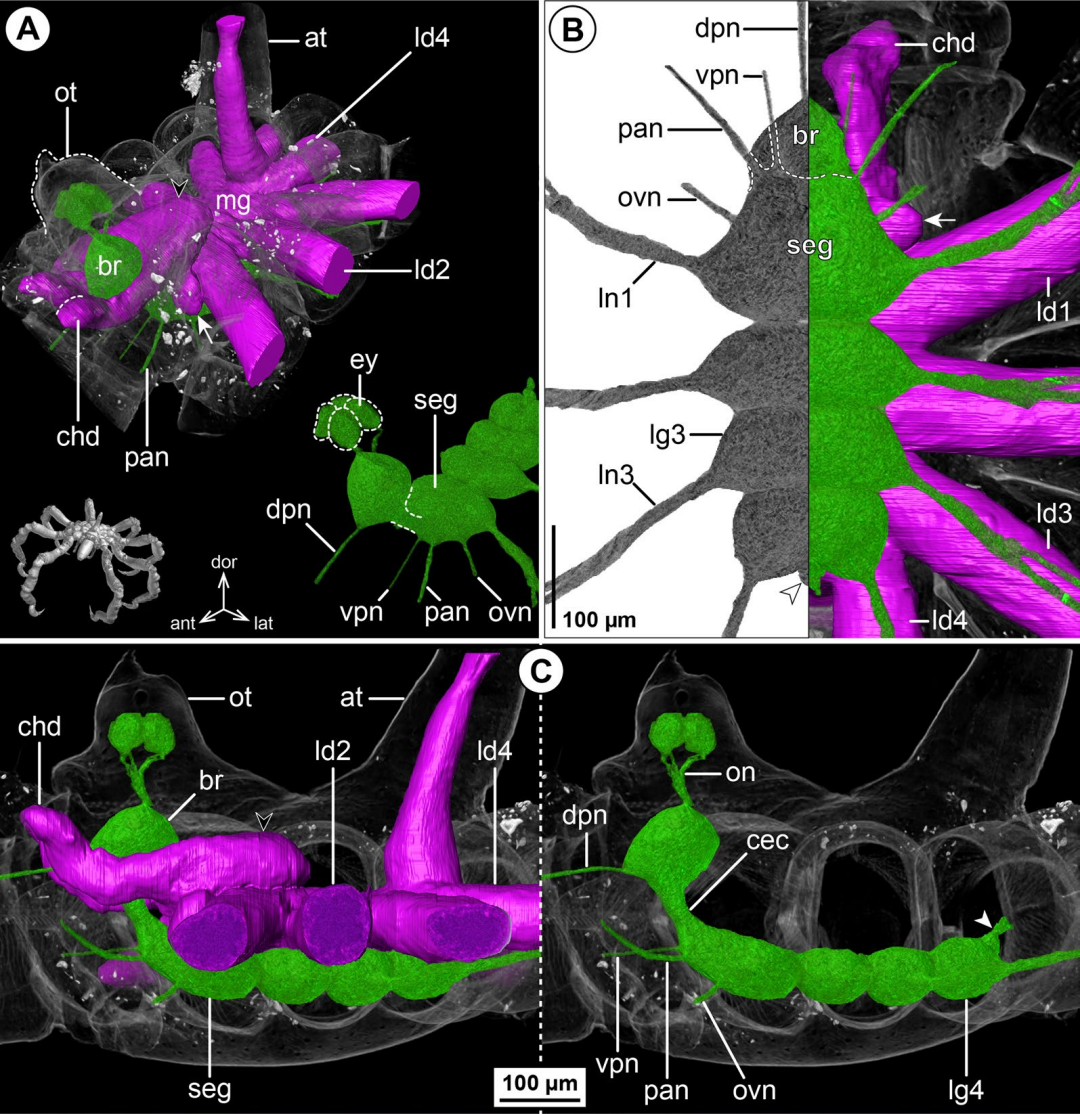
The central nervous system and midgut in the trunk of *Tanystylum orbiculare* (Ammotheidae). Reconstructions of the CNS (3D volume rendering, green) and midgut (3D surface, magenta) based on a µCT scan of an adult male. The white arrowheads point to the origin of the posteriorly projecting proctodeal nerve. The white arrows indicate a small lateral gut protrusion anterior to leg diverticulum 1. The black arrowheads mark the dorsal pouch of the midgut. For better pattern visualization, all major nerves of the right body half were virtually removed in **A** (right side) and **C**. **A** Oblique antero-lateral view. Left bottom corner: overview of the specimen. Top: complete CNS and midgut reconstruction. Right bottom corner: CNS and eyes. **B** Ventral view. Left side: CNS in grayscale. Right side: CNS and midgut. For reference, unreconstructed dorsal parts of the trunk are shown in transparent gray. **C** Lateral view of the CNS with and without the midgut structures (left and right, respectively). For reference, unreconstructed parts of the right body half are shown in transparent gray

The ammotheid genus *Tanystylum* is characterized by an extremely compact, disc-shaped trunk (Fig. 10A). The CNS is likewise very compact and all ventral ganglia are touching (Fig. 10B, C). The palpal, ovigeral and leg 1 neuromeres form an extended SEG with one contiguous soma cortex and a fused neuropil core (Fig. 10B, C; Additional file 2: Fig. S1E). While a CHN to the vestigial cheliphores could not be traced in the µCT data, all other major cephalic nerves (DPN, VPN, PAN, OVN) were identified (Fig. 10A, C). ISNs are missing (Fig. 10B, Additional file 4: Fig. S3E), correlating with the complete fusion of the trunk segments and the complete absence of longitudinal trunk musculature.

The midgut tube is very short and starts to angle steeply upward into the erect anal tubercle at the level of the lateral processes of the fused trunk segment 3 (Fig. 10A, C). All LDs arise next to each other, with LD4 emerging at the presumptive border of the fused trunk segments 3 and 4, directly anterior to the posteriorly directed lateral process of leg 4 (Fig. 10B). Similar to *A. echinata*, the lumen of the LDs reaches the tibia 2-tarsus border. A CHD to the vestigial cheliphore is present (Fig. 10A–C) and an additional small lateral pouch protrudes directly anterior to LD1 (Fig. 10A, B). Remarkably, at the point where the esophagus opens into the midgut, the latter features a dorsally protruding, widened portion directly posterior to the brain (Fig. 10A, C).

#### Additional ammotheid representatives studied

Low-resolution µCT scans of two species of the genus *Ammothea* revealed the full number of ventral ganglia (Additional file 5: Fig. S4D, E). In the more attenuate *A. clausi* Pfeffer, 1889, the ventral ganglia are interconnected by short connectives (Additional file 5: Fig. S4D), whereas in a more compact, undetermined *Ammothea* sp., all ventral ganglia are touching (Additional file 5: Fig. S4E). A similar situation is found in the genus *Ammothella*, in which the more elongated *A. biunguiculata* (Dohrn, 1881) features inter-ganglionic connectives (Additional file 5: Fig. S4F), while the VNC of the minute, compact *A. longipes* (Hodge, 1864) shows touching but separate ganglia (Additional file 5: Fig. S4G).

### Colossendeidae

#### ***Colossendeis angusta*** Sars, 1877; Fig. 11

**Fig. 11:**
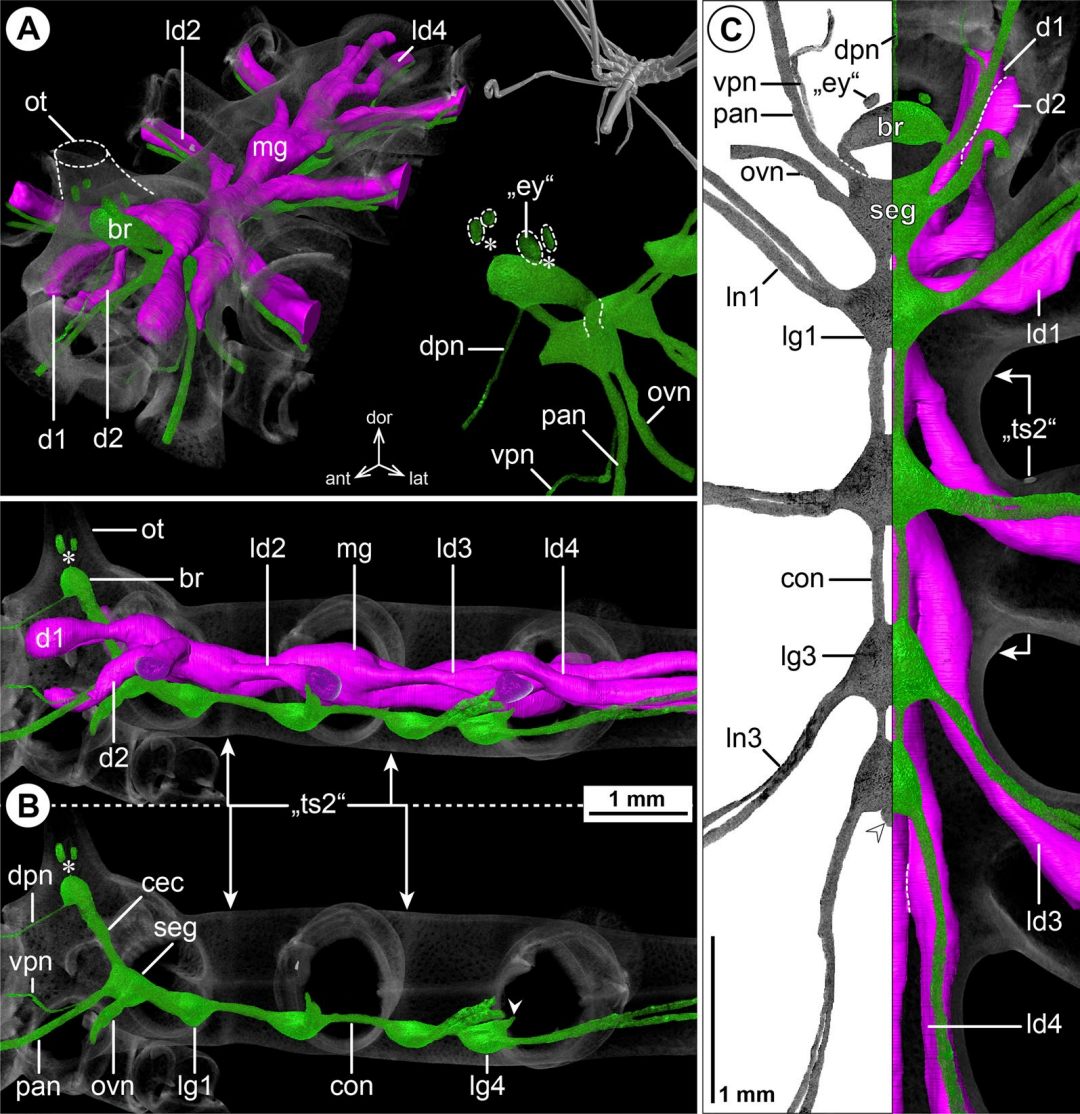
The central nervous system and midgut in the trunk of *Colossendeis angusta* (Colossendeidae). Reconstructions of the CNS (3D volume rendering, green) and midgut (3D surface, magenta) based on a µCT scan of an adult female. The white arrowheads point to the origin of the posteriorly projecting proctodeal nerve. Asterisks indicate the incompletely reconstructed optic nerve (insufficient resolution). Trunk segment 2 is labeled in quotation marks (“ts2”) as it is fused with the other segments, but fusion borders are still discernible in the cuticle. For better pattern visualization, all major nerves of the right body half were virtually removed in **A** (right side) and **B**. **A** Oblique antero-lateral view. Right top corner: overview of the specimen. Left side: complete CNS and midgut reconstruction. Right bottom corner: anterior portion of the CNS and eyes. **B** Lateral view of the CNS with and without the midgut structures (top and bottom, respectively). For reference, unreconstructed parts of the right body half are shown in transparent gray. **C** Ventral view. Left side: CNS in grayscale. Right side: CNS and midgut. Note the two anterior midgut diverticula (d1 and d2). For reference, unreconstructed dorsal parts of the trunk are shown in transparent gray

The brain is located far dorsally, extending into the base of the ocular tubercle (Fig. 11B). Although *C. angusta* lacks externally discernible eyes, vestigial eye anlagen are identifiable below the ocular tubercle’s thick cuticle (Fig. 11A, B). Optic nerves could not be traced in the µCT scans. The medio-lateral axis of the brain is elongated, giving it a shape of a transversally orientated and slightly downward-curved cone (Fig. 11A, C) from which the unusually long CEC emerges ventro-laterally (Fig. 11A, B). A CHN could not be identified. Apart from a forward shift of LG4 all the way to the center of trunk segment 3, the arrangement of the ventral ganglia shows no remarkable features (Fig. 11B, C). Notably, the VPN does not have a separate nerve root, but emerges from the SEG together with the PAN, to branch off at some distance and project into the proboscis (Fig. 11A, B). The leg nerves split into two main branches in the lateral processes (Fig. 11A,C). Intersegmental nerves were not found, correlating with the complete lack of ventral longitudinal musculature in the fully fused trunk segments (Fig. 11B, C).

The midgut features two anteriorly projecting diverticula (Fig. 11). One of them extends to the dorso-lateral proboscis base, reminiscent of a CHD (although cheliphores are completely reduced in *Colossendeis* adults) (Fig. 11A, B). The other diverges near the SEG from the central midgut (Fig. 11C), corresponding in position to the small lateral protrusions in Endeidae, Pallenopsidae and Ascorhynchidae. In *C. angusta*, however, this diverticulum extends further toward the base of the prominent palp. In contrast to all other families hitherto described, the divergence points of LD2 to LD4 are noticeably forward-shifted, lying far anterior to the lateral processes they target, and in the case of LD3 and LD4 even in the next anterior trunk segment (Fig. 11B, C). Owing to the large size of *C. angusta*, the distal leg articles were not included in the µCT scans and the extension of the LD lumen could not be traced beyond the femur.

### Pycnogonidae

#### ***Pycnogonum litorale*** (Strøm, 1762); Fig. 12

**Fig. 12:**
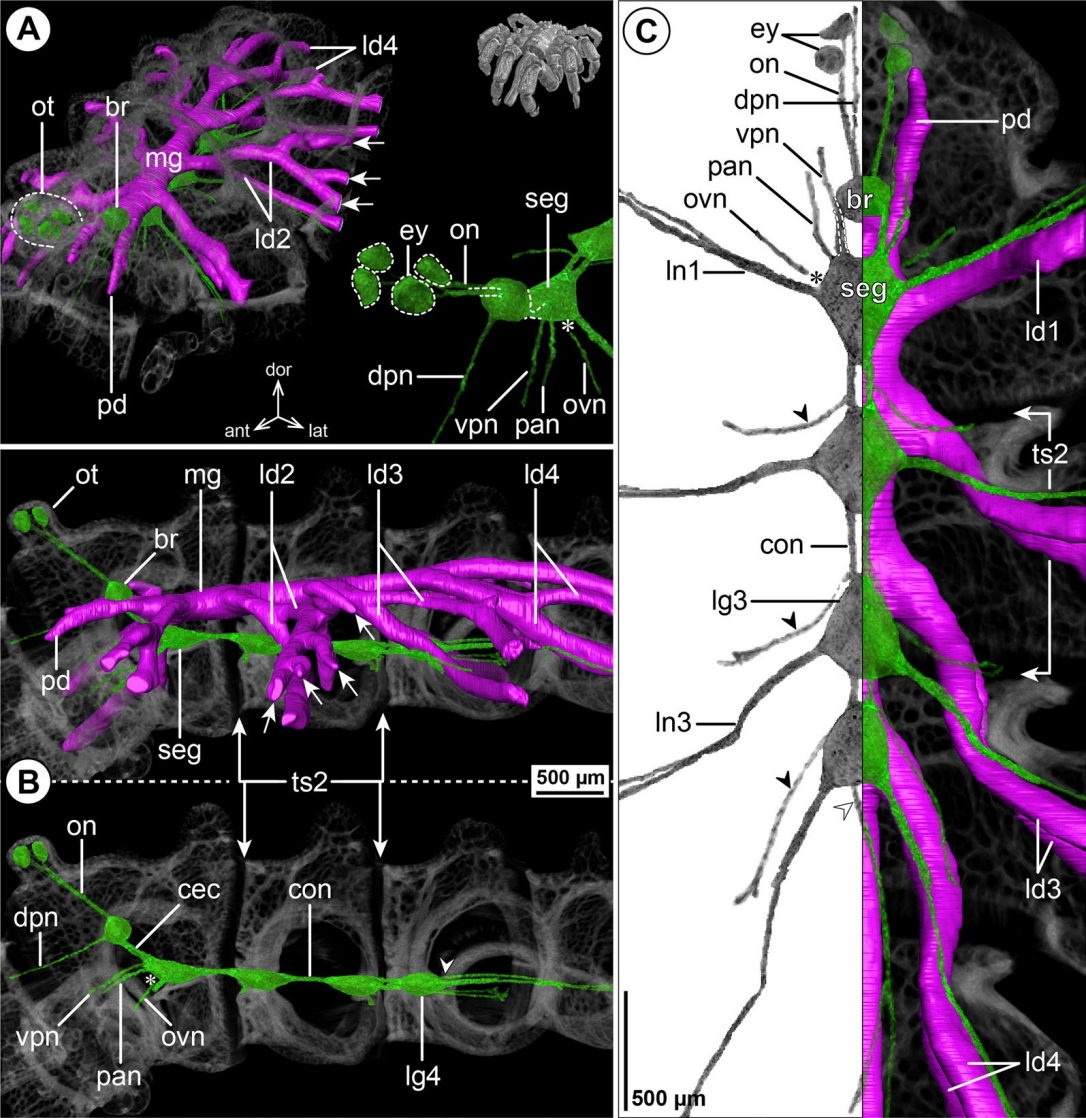
The central nervous system and midgut in the trunk of *Pycnogonum litorale* (Pycnogonidae). Reconstructions of the CNS (3D volume rendering, green) and midgut (3D surface, magenta) based on a µCT scan of an adult male. The white arrowheads point to the origin of the posteriorly projecting proctodeal nerve. Asterisks indicate an incompletely reconstructed portion of the ovigeral nerve (tissue damage). The white arrows highlight additional subdivisions of the dorsal branch of leg diverticulum 2. For better pattern visualization, all major nerves of the right body half were virtually removed in **A** (right side) and **B**. **A** Oblique antero-lateral view. Right top corner: overview of the specimen. Left side: complete CNS and midgut reconstruction. Right bottom corner: anterior portion of the CNS and eyes. **B** Lateral view of the CNS with and without the midgut structures (top and bottom, respectively). For reference, unreconstructed parts of the right body half are shown in transparent gray. **C** Ventral view. Left side: CNS in grayscale. The black arrowheads point to the intersegmental nerves. Right side: CNS and midgut. For reference, unreconstructed dorsal parts of the trunk are shown in transparent gray

In this robust species without cheliphores and palps, the brain is located centrally in the cephalosoma and connected to the SEG by a long soma-free CEC (Fig. 12B). The optic nerve is longer than in most other families, as the ocular tubercle arises at the antero-dorsal margin of the cephalosoma (Fig. 12A, B). A CHN could not be traced in the µCT data. The SEG is extended, encompasses the palpal, ovigeral and leg 1 neuromeres (Fig. 12B, C; Additional file 2: Fig. S1F) and displays the full suite of major nerves (VPN, PAN, OVN, LN1), in spite of the lack of palps and—in females—of ovigers (Fig. 12B, C; Additional file 2: Fig. S1F). The remaining ventral ganglia are interconnected by soma-free connectives from which ISNs emanate (Fig. 12C). Compared to all families hitherto described, the ventral ganglia show a more pronounced forward shift; LG3 is completely included in trunk segment 2 and LG4 is located in the anterior half of trunk segment 3 (Fig. 12B, C).

The midgut exhibits several deviations from the basic layout. A short diverticulum extends anteriorly into the proboscis base (Fig. 12; Additional file 7: Fig. S6B, B’). Apart from LD1, all LDs branch off the midgut distinctly anterior to the lateral processes they target. While LD2 emerges at the border of cephalosoma and trunk segment 2, LD3 and LD4 are even more forward-shifted into trunk segments 2 and 3, respectively (Fig. 12B, C; Additional file 7: Fig. S6B’), reminiscent of the pattern observed in Colossendeidae. Most strikingly, however, each LD bifurcates into a ventral and a dorsal branch near the base of its lateral process (Fig. 12A, B). While the ventral branch extends all the way into the tarsus, the dorsal branch terminates in the femur. Beyond that, the dorsal branch features additional shorter offshoots as it projects distally (Fig. 12A, B; Additional file 7: Fig. S6B).

#### ***Pycnogonum gaini*** Bouvier, 1910; Fig. 13

**Fig. 13:**
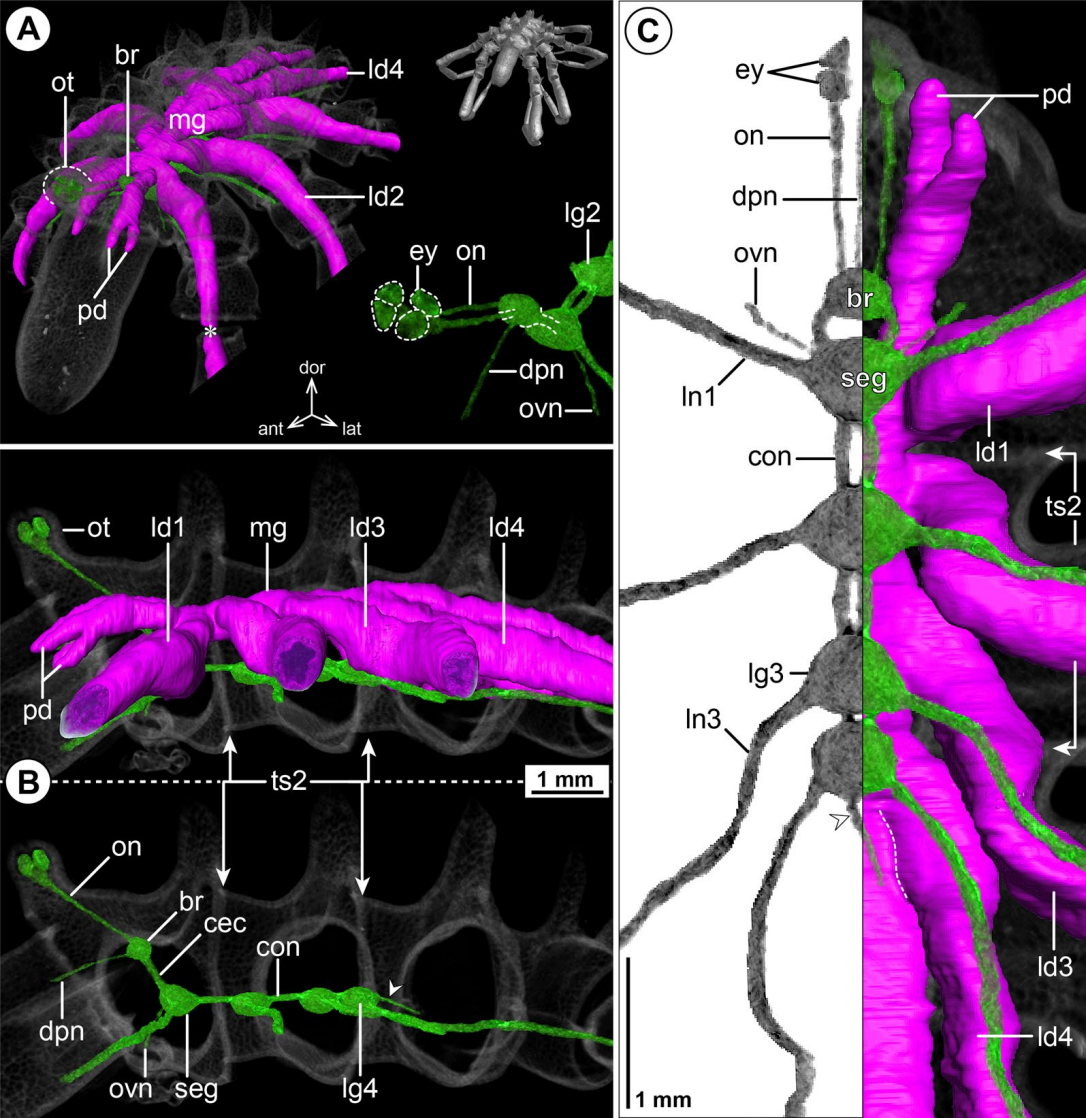
The central nervous system and midgut in the trunk of *Pycnogonum gaini* (Pycnogonidae). Reconstructions of the CNS (3D volume rendering, green) and midgut (3D surface, magenta) based on a µCT scan of an adult male. The white arrowheads point to the origin of the posteriorly projecting proctodeal nerve. For better pattern visualization, all major nerves of the right body half were virtually removed in **A** (right side) and **B**. **A** Oblique antero-lateral view. Right top corner: overview of the specimen. Left side: complete CNS and midgut reconstruction. Note the absence of additional bifurcations of the leg diverticula. The asterisk indicates an incompletely reconstructed portion of leg diverticulum 1 (tissue damage). Right bottom corner: anterior portion of the CNS and eyes. **B** Lateral view of the CNS with and without the midgut structures (top and bottom, respectively). For reference, unreconstructed parts of the right body half are shown in transparent gray. **C** Ventral view. Left side: CNS in grayscale. Right side: CNS and midgut. For reference, unreconstructed dorsal parts of the trunk are shown in transparent gray

The layout of the CNS in *P. gaini* is very similar to the one in *P. litorale*. This includes the extended SEG and the significant forward shift of the ventral ganglia, with LG4 located even more anteriorly, at the border of trunk segments 2 and 3 (Fig. 13B, C). The presence of several major nerves (CHN, VPN, PAN and ISNs) could not be reconstructed with confidence (Fig. 13), which was likely due to the lower optical magnification and correspondingly lower structural resolution in the µCT scan of this larger representative.

The divergence points of the midgut LDs from the central tube conform with the forward-shifted pattern in *P. litorale* (Fig. 13B, C). However, they do not feature additional bifurcations but resemble linear but very voluminous tubes (Fig. 13A, B; Additional file 7: Fig. S6C, C’) that reach into the tarsus, in correspondence to the ventral LD branch in *P. litorale*. The proboscis diverticulum of *P. gaini* splits at the proboscis base into a dorsal and a ventral branch (Fig. 13A, B; Additional file 7: Fig. S6C), reminiscent of the pattern observed in Endeidae (see Fig. 6A).

#### Additional pycnogonid representatives studied

The CNS of the very large *Pycnogonum diceros* Marcus, 1940 shares its basic layout with *P. litorale* and *P. gaini* (Additional file 8: Fig. S7), indicative of a high degree of size-independent conservation within the family. Notably, the anterior shift of the ventral ganglia is even more pronounced than in *P. gaini* and as a result, LG4 is completely included in trunk segment 2 (Additional file 8: Fig. S7B, C). Owing to the lower optical resolution and the comparatively low contrast of nerves in the surrounding connective tissue, the presence of several major nerves (CHN, VPN, OVN and ISNs) could not be unambiguously confirmed in the µCT scan. While a putative PAN was reconstructed (Additional file 8: Fig. S7), its exact target region remains unclear. The leg nerves of LG2 to LG4 each feature a first bifurcation before entering their respective lateral process: the stronger branch represents the leg nerve proper and continues into its respective lateral process, the more delicate second branch projects toward the body wall and could not be traced further with certainty (Additional file 8: Fig. S7C).

The midgut branching pattern is more complex than in any other pycnogonid hitherto investigated. Similar to *P. gaini* and *E. spinosa*, the proboscis diverticulum displays a bifurcation (Additional files 7, 8: Figs. S6D, D’; S7A, B). The LDs emerge closely spaced from the central tube with divergence points that are even more forward-shifted than in *P. litorale* and *P. gaini*. As a consequence, the ultimate LD4 branches off already in trunk segment 2 (Additional files 7, 8: Figs. S6D’; S7C). Notably, a one-sided, secondary connection between the posteriorly extending right LD4 and the central tube was found (Additional file 7: Fig. S6D’). Each leg diverticulum bifurcates more than once in the trunk, leading to a voluminous network of midgut branches (Additional files 7, 8: Figs. S6D, D’; S7B). Among others, a short dorsal sub-branch of each LD projects back in a medial direction, running anterior to the segment border beneath the dorsal integument (Additional files 7, 8: Figs. S6D; S7A). Additional irregular branching occurs within the leg up to the distal end of tibia 1, from where one tube extends to the end of tibia 2. In addition, the central midgut features two prominent median projections that extend into the unpaired dorso-median tubercles of the cephalosoma and trunk segment 2 (Additional files 7, 8: Figs. S6D; S7A, B).

### Rhynchothoracidae

#### ***Rhynchothorax australis*** Hodgson, 1907; Fig. 14

**Fig. 14:**
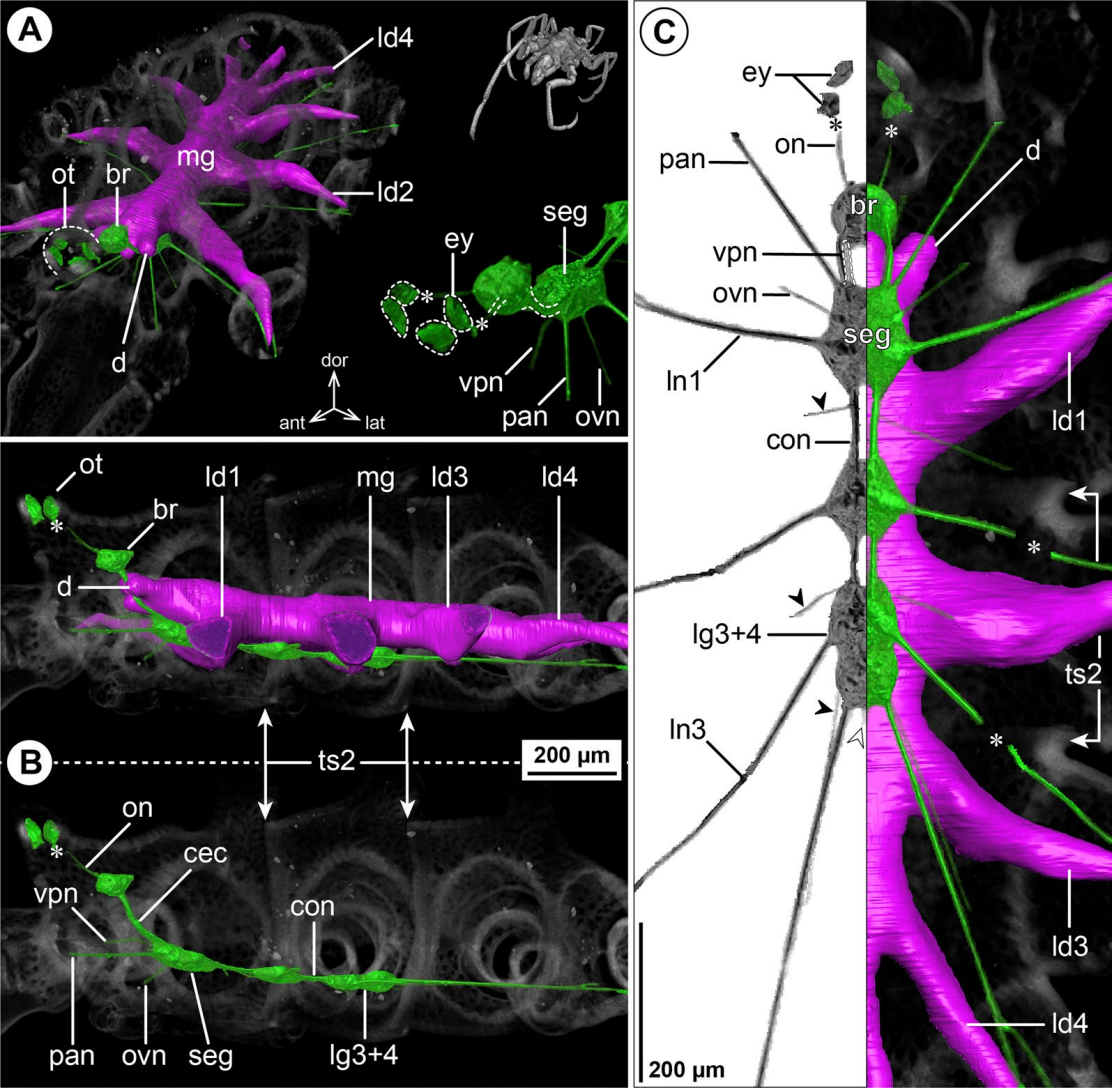
The central nervous system and midgut in the trunk of *Rhynchothorax australis* (Rhynchothoracidae). Reconstructions of the CNS (3D volume rendering, green) and midgut (3D surface, magenta) based on a µCT scan of an adult female. Asterisks indicate incompletely reconstructed portions of the optic nerve (insufficient resolution) and damaged regions of leg nerves. For better pattern visualization, all major nerves of the right body half were virtually removed in **A** (right side) and **B**. **A** Oblique antero-lateral view. Right top corner: overview of the specimen. Left side: complete CNS and midgut reconstruction. Right bottom corner: anterior portion of the CNS and eyes. **B** Lateral view of the CNS with and without the midgut structures (top and bottom, respectively). For reference, unreconstructed parts of the right body half are shown in transparent gray. **C** Ventral view. Left side: CNS in grayscale. The black arrowheads point to the intersegmental nerves. The white arrowhead indicates the origin of the posteriorly projecting proctodeal nerve. Right side: CNS and midgut. For reference, unreconstructed dorsal parts of the trunk are shown in transparent gray

Although the relatively compact Rhynchothoracidae are of small body size, the CNS does not take up as much space in the body cavity as in similarly small representatives of other families (compare Fig. 14 and Figs. 4, 8, 9). The dorso-ventrally flattened brain is located in the anterior half of the cephalosoma and connected to the SEG by a long soma-free CEC (Fig. 14B). The DPN and CHN could not be confidently traced in the µCT data (Fig. 14A, B), but tubulin immunolabeling of CNS whole mounts confirms their presence, although the nerve corresponding to the CHN (the cheliphore is lacking in adults) is very delicate (not shown). The SEG includes the palpal, ovigeral and leg 1 neuromeres and features the full set of major nerves (VPN, PAN, OVN, LN1) (Fig. 14A, C; Additional file 2: Fig. S1D). While soma-free connectives are formed between the SEG to LG3, the latter is incompletely or even completely fused to LG4, depending on specimen studied (Fig. 14C; Additional file 4: Fig. S3G, H). The ventral ganglia are forward-shifted to the extent that LG3 + 4 is fully included in trunk segment 2 (Fig. 14B, C). All ISNs are present (Fig. 14C). Notably, the last ISN has its root between the partially or completely fused LG3 and LG4 and extends through the soma cortex of the latter before projecting posteriorly, in parallel to the leg nerve of LG4 (Fig. 14C; Additional file 4: Fig. S3G, H).

The midgut features a small, anteriorly protruding diverticulum that does not extend past the brain (Fig. 14B, C). The divergence point of the LD3 lies in the anterior half of trunk segment 3, slightly anterior to its lateral process (Fig. 14C). Leg diverticulum 4 displays a more pronounced anterior shift, emerging in the posterior half of trunk segment 3 (Fig. 14C). The lumen of each LD is very small, as it narrows to a compact tissue strand after having passed through the lateral process into coxa 1 (Fig. 14A, C).

### Austrodecidae

#### ***Austrodecus glaciale*** Hodgson, 1907; Fig. 15

**Fig. 15:**
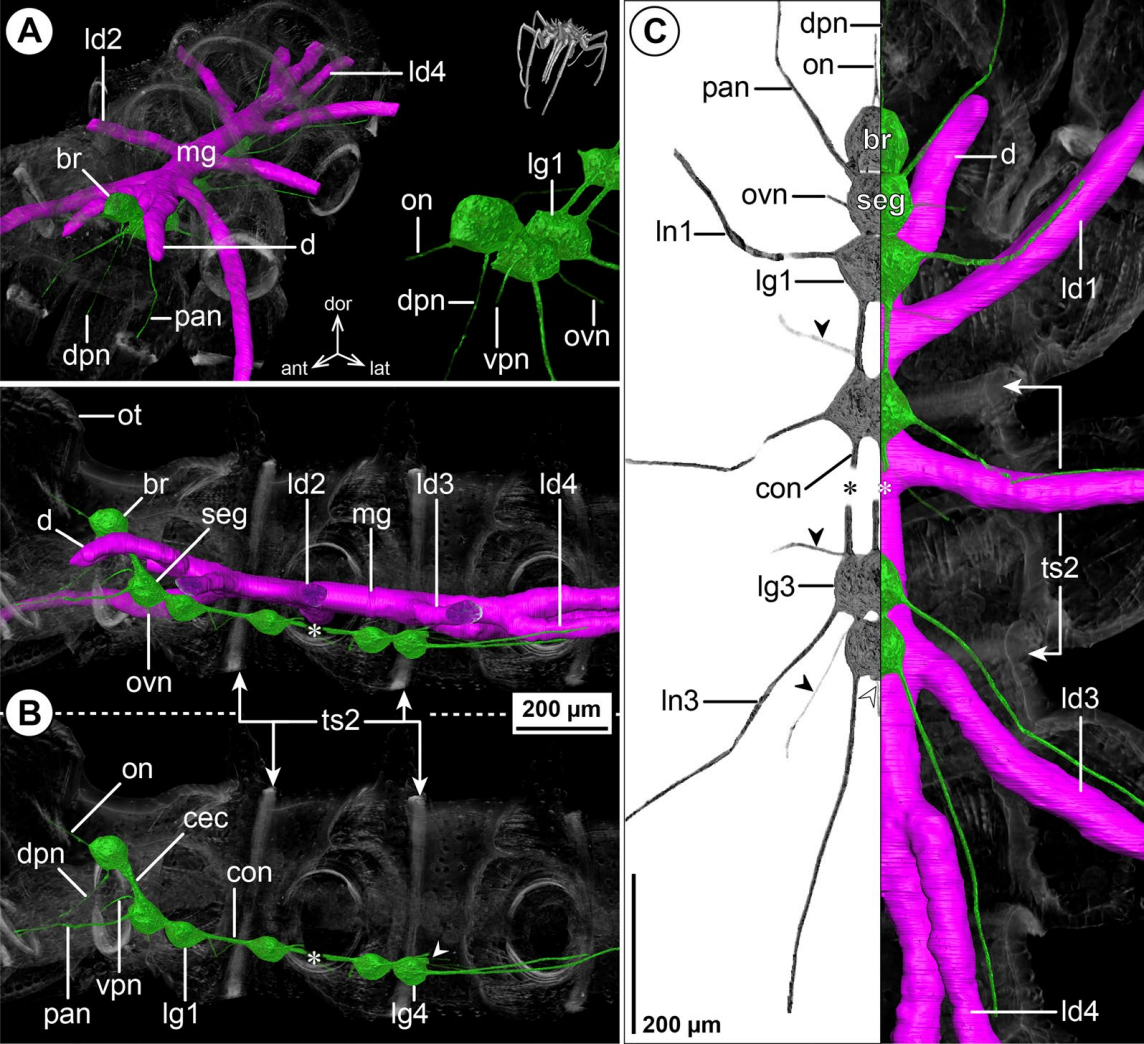
The central nervous system and midgut in the trunk of *Austrodecus glaciale* (Austrodecidae). Reconstructions of the CNS (3D volume rendering, green) and midgut (3D surface, magenta) based on a µCT scan of an adult female. The white arrowheads indicate the origin of the posteriorly projecting proctodeal nerve. Asterisks indicate damaged connectives. Note that the eyes are not included in the scan, owing to the extreme elongation of the ocular tubercle. For better pattern visualization, all major nerves of the right body half were virtually removed in **A** (right side) and **B**. **A** Oblique antero-lateral view. Right top corner: overview of the specimen. Left side: complete CNS and midgut reconstruction. Right bottom corner: anterior portion of the CNS and eyes. **B** Lateral view of the CNS with and without the midgut structures (top and bottom, respectively). For reference, unreconstructed parts of the right body half are shown in transparent gray. **C** Ventral view. Left side: CNS in grayscale. The black arrowheads point to the intersegmental nerves. Right side: CNS and midgut. For reference, unreconstructed dorsal parts of the trunk are shown in transparent gray

Similar to *R. australis*, the CNS of the small *A. glaciale* does not occupy as much space in the body cavity as in species of comparable size from other families. The spherical brain is connected to the SEG by a long CEC (Fig. 15B). The paired optic nerve converges with its contralateral counterpart at a short distance from the brain to project into the extremely elongated, antero-dorsally directed ocular tubercle (Fig. 15A, C). A CHN could not be traced in the µCT scans studied. The SEG and LG1 are touching but unfused and show the full complement of major nerves (Fig. 15B, C; Additional file 2: Fig. S1B). The PAN is far more prominent than the OVN (Additional file 2: Fig. S1B), which correlates with the marked size differences of the two appendages. The remaining VNC ganglia are forward-shifted; LG4 is located between trunk segments 2 and 3 (Fig. 15B, C). All ISNs are present (Fig. 15C; Additional file 4: Fig. S3F).

The midgut sends a short anterior diverticulum past the brain toward the bases of the proboscis and palps (Fig. 15). The divergence points of LD3 and LD4 are forward-shifted in relation to their lateral processes, lying at the border to (LD3) or within (LD4) the next anterior trunk segment (Fig. 15C). The lumen of the LDs ends in the femur.

#### Additional austrodecid representatives studied

To assess whether the forward-shifted VNC is characteristic of the entire family, a subadult of *Pantopipetta armoricana* Stock, 1978, a member of the slenderer, second austrodecid genus, was studied (Additional file 9: Fig. S8). In this species, the positions of the VNC ganglia closely resemble the nymphonid pattern, with LG3 and LG4 located in trunk segment 3 (Additional file 9: Fig. S8B, C), which demonstrates the presence of intra-familial variability in this regard. Further, none of the midgut LDs shows a forward shift, their divergence points from the central tube aligning with the lateral process of the respective trunk segments (Additional file 9: Fig. S8A, C; LD4 not shown). As in *Austrodecus glaciale*, the lumen of each LD does not extend beyond the femur. At the midgut’s anterior end, a tiny protrusion may correspond in position to the more prominent anterior diverticulum of *A. glaciale* (Additional file 9: Fig. S8A, B).

## Discussion

### An anteriorly shifted VNC as an ancestral feature of crown group Pycnogonida?

**Fig. 16:**
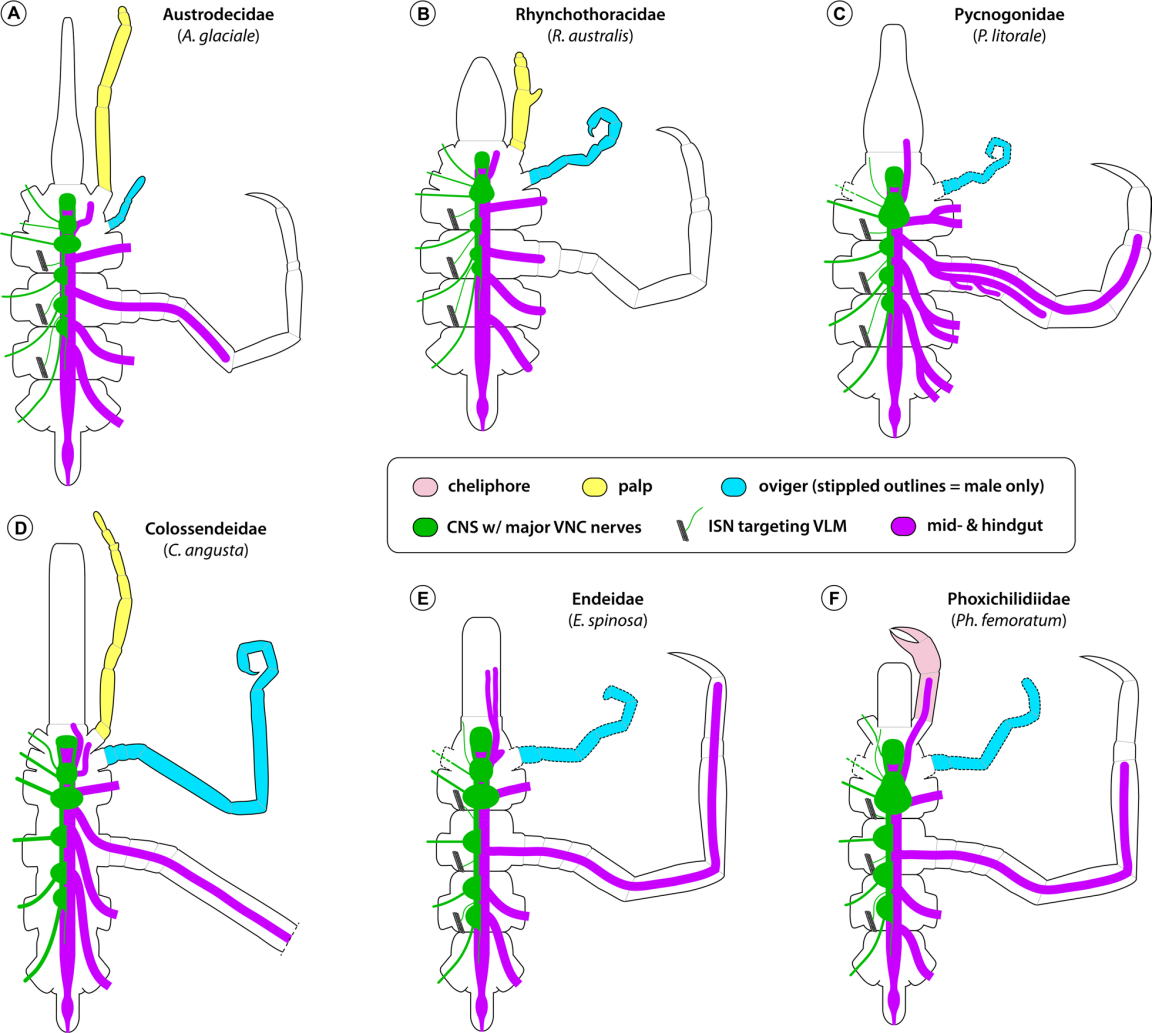
Central nervous system and midgut layout in various pycnogonid families in relation to external morphology. Schematized ventral views, anterior to the top. The configuration of the cephalic limbs (cheliphore—light red; palp—yellow; oviger—light blue) and the second leg are shown in the left body half. The CNS ganglia (including the anterior brain) are depicted in green and starting from leg ganglion 2, are shown only in the right body half. Nerves are likewise depicted in the right half only, with optic and proboscis nerves completely omitted. The identity of the ganglia can be deduced from the segment and appendage they innervate. The midgut and hindgut are depicted in magenta, the extension of the leg diverticula is exemplarily illustrated in the second leg. **A** Austrodecidae—*A. glaciale*. **B** Rhynchothoracidae—*R. australis*. **C** Pycnogonidae—*P. litorale*. **D** Colossendeidae—*C. angusta* (leg 2 shown up to the femur only). **E** Endeidae—*E. spinosa*. **F** Phoxichilidiidae—*Ph. femoratum*

**Fig. 17:**
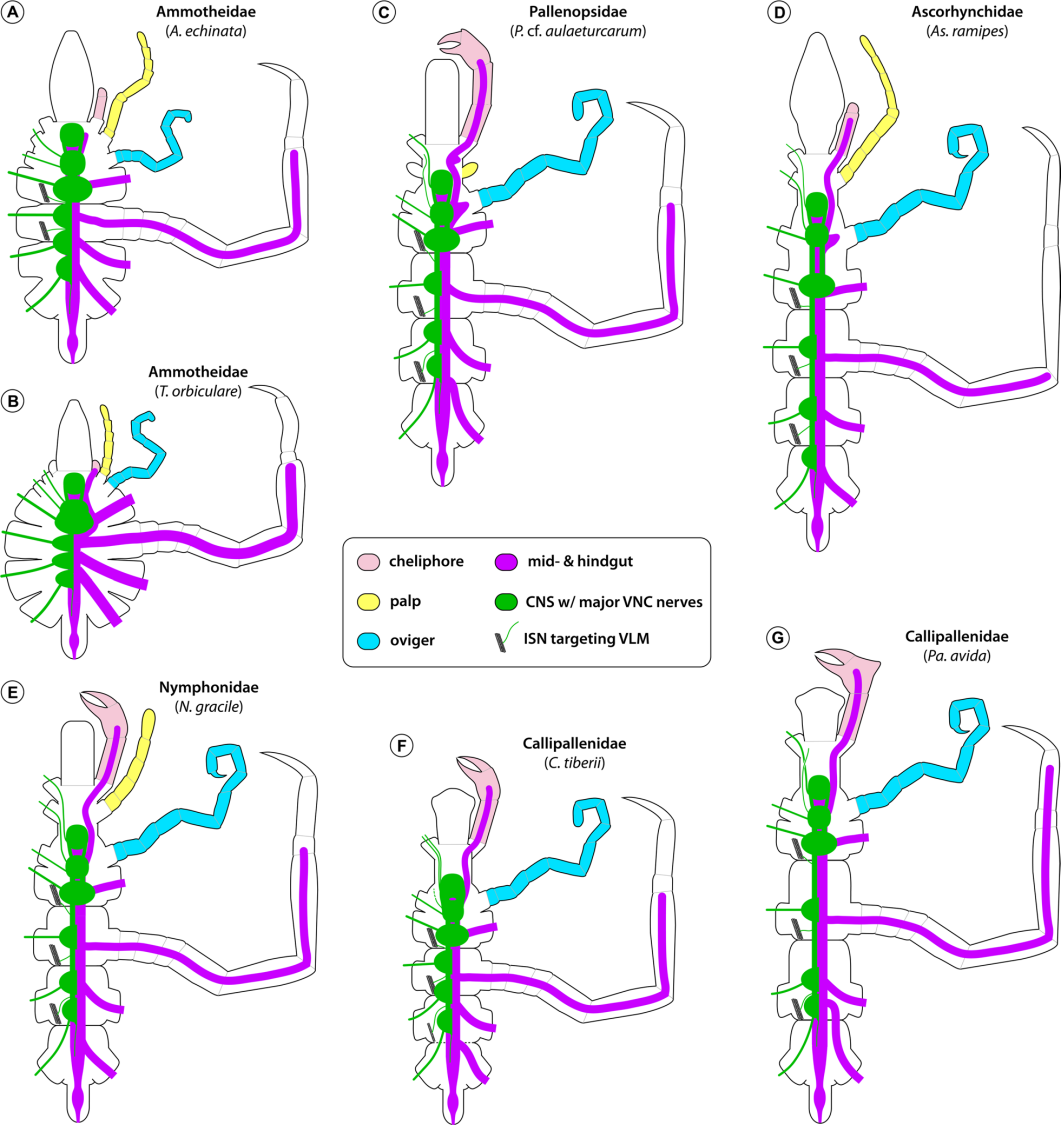
Central nervous system and midgut layout in various pycnogonid families in relation to external morphology (continued). Schematized ventral views, anterior to the top. The configuration of the cephalic limbs (cheliphore—light red; palp—yellow; oviger—light blue) and the second leg are shown in the left body half. The CNS ganglia (including the anterior brain) are depicted in green and starting from leg ganglion 2, are shown only in the right body half. Nerves are likewise depicted in the right half only, with optic and proboscis nerves completely omitted. The identity of the ganglia can be deduced from the segment and appendage they innervate. The midgut and hindgut are depicted in magenta, the extension of the leg diverticula is exemplarily illustrated in the second leg. **A** Ammotheidae– *A. echinata*. **B** Ammotheidae—*T. orbiculare*. **C** Pallenopsidae—*P.* cf. *aulaeturcarum*. **D** Ascorhynchidae—*As. ramipes*. **E** Nymphonidae—*N. gracile*. **F** Callipallenidae—*C. tiberii.* **G** Callipallenidae—*Pa. avida*

**Fig. 18:**
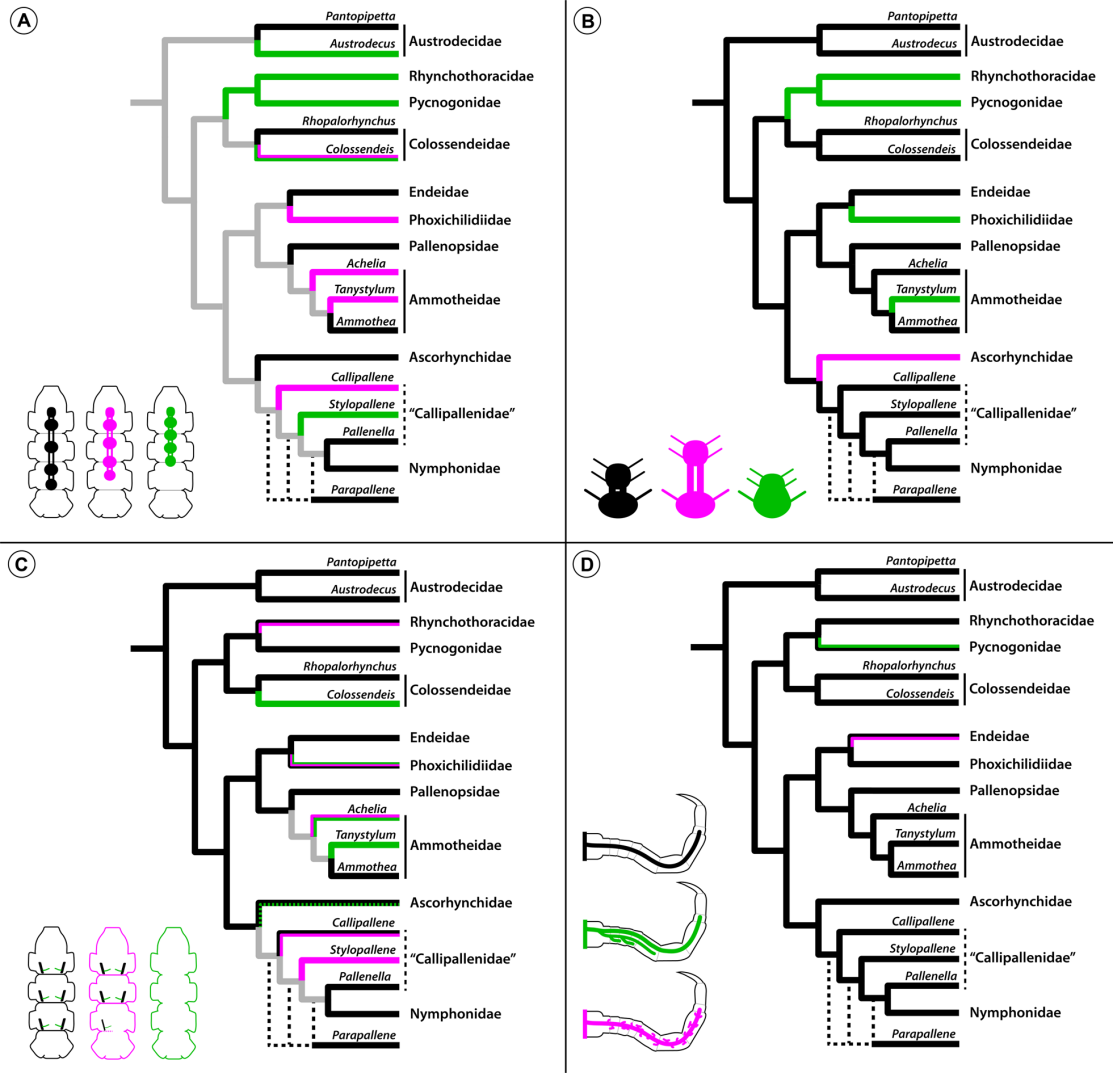
Mapping of CNS and midgut characters on pycnogonid phylogeny. The depicted cladogram has been simplified from [12]. Three characters of the CNS (**A**–**C**) and one character pertaining to the midgut diverticula (**D**) are mapped, colored branches represent the different character states schematically depicted in the bottom left corner of each cladogram. In families shown as one collapsed lineage, intra-familial variation of a character is indicated by side-by-side depiction of its different states. Owing to the lack of a well-supported phylogeny and insufficient neuroanatomical sampling at the genus level, transformations along the branches of the morphologically diverse families Ammotheidae and “Callipallenidae” are considered ambiguous (gray) in case of multiple intra-familial character states (**A**, **C**). The stippled green line in **C** relates to the potential inclusion of the genus *Paranymphon* in the Ascorhynchidae, as indicated in [12]. **A** Antero-posterior location of leg ganglia: LG3&4 in TS3 (black); LG3 at TS2-3 border (magenta); LG3(&LG4) in TS2 (green). **B** SEG and LG1: close but anatomically separate (black); set off with distinct connectives (magenta); fused into extended SEG (green). **C** ISNs and trunk segmentation: complete ISN set in fully segmented trunk (black); reduced ISN set in partially fused trunk (pink); lack of ISNs in completely fused trunk (green). **D** Structure of leg diverticula: linear (black); multi-branching (green); linear with irregular pouches (magenta)

An anterior shift of ventral ganglia relative to the external trunk segment borders has been highlighted in previous descriptions of pycnogonid anatomy [43, 46, 47]. In this context, the location of the ultimate LG4 within the posterior half of trunk segment 3 has been recognized as a widespread pattern present in many of the families most frequently investigated, such as Nymphonidae, Callipallenidae and Endeidae (Figs. 16E; 17E, G). Beyond that, also the more pronounced anterior shift of the VNC in genera of Colossendeidae and Pycnogonidae was previously reported [15, 26, 43, 46, 47] and recently confirmed in *Pycnogonum litorale* [69] (Fig. 16C, D). Loman [47] interpreted the A–P distribution of the LGs and the inter-ganglionic distances as being strictly correlated with the A–P extensions of the trunk segments. This notion may be readily applied to the minute and compact representatives of genera such as *Tanystylum*, *Achelia*, *Callipallene* or *Anoplodactylus*, in which the closely packed LGs occupy the majority of limited space in the body cavity’s ventral half (Fig. 17A, B, F; see [26, 43, 59] for further examples), or to more elongated forms, as represented by *Parapallene* or *Ascorhynchus* in this study (Fig. 17D, G; see [46, 59, 61] for further examples). However, in none of the families with the most pronounced anterior shift of the VNC (members of Austrodecidae, Rhynchothoracidae, Pycnogonidae, Colossendeidae), limited body cavity space appears to be an issue. Although the VNC terminates already near the border of trunk segments 2 and 3, the LGs are not even maximally compressed along the A–P axis, as evidenced by the presence of soma-free connectives (Fig. 16A–D). Also general body size cannot account for this phenomenon, as austrodecids and rhynchothoracids are among the smallest pycnogonid representatives, whereas Pycnogonidae and Colossendeidae comprise medium-sized to very large forms. Moreover, our study of three differently sized members of the genus *Pycnogonum* does not show any distinct size-related trends in terms of the VNC’s A–P extension. These observations raise the question whether an anterior condensation of the LGs may represent a phylogenetically informative trait. According to the best supported pycnogonid phylogeny available [12], the four families form a grade subtending the rest of the pycnogonid lineages (Fig. 18), which is suggestive of an anteriorly condensed VNC as plesiomorphic trait. Some support for the derived nature of further posteriorly shifted LG3 and LG4 in other groups may also be seen in the VNC development in families like Nymphonidae, Callipallenidae and Endeidae [10, 43, 70, 71]. Here, the originally closely spaced LG anlagen start to separate along the A–P axis only during late postembryonic development, in parallel to the growth and elongation of the trunk segments. By contrast, the LG anlagen of *P. litorale*—the only species of the basal grade for which ontogenetic data are available—retain their more anterior location during postembryonic trunk growth [69]. However, the scenario of a secondary posterior shift of LGs within the pycnogonid crown group is challenged by intra-familial pattern variability in two of the four basally branching families (Fig. 18A). In the very slender austrodecid genus *Pantopipetta* ([46, 61]; this study) and in the extremely delicate colossendeid genus *Rhopalorhynchus* [46], LG3 and LG4 are found together in trunk segment 3. These examples rehabilitate Loman’s view [46] to some extent, only that not trunk elongation alone (typically assessed by distances between lateral processes), but perhaps more importantly the extreme medio-lateral narrowing of segments seem to influence the position of the VNC ganglia. A possible physiological factor counteracting a more anterior VNC condensation in extremely attenuate forms may be the negative impact on hemolymph circulation through the ventral body cavity if all ganglia were concentrated in the anterior body half.

Owing to the intra-familial pattern variability within the basal grade, the pronounced anterior shift of the VNC may be only confidently traced to the last common ancestor of Pycnogonidae and Rhynchothoracidae (Fig. 18A). In contrast to a previous claim [15], all data available for these two families consistently recover this feature ([26, 43, 68, 69]; this study). Notably, also in the remaining parts of the phylogeny, the A–P distribution of the LGs does not display stable patterns at the family level (e.g., Ammotheidae, Callipallenidae) (Fig. 18A), which further questions its suitability for phylogenetic inference of inter-familial relationships.

### The extended tripartite SEG supports Rhynchothoracidae + Pycnogonidae

Beyond its location relative to the external segment borders, the adult CNS of several families displays actual anatomical fusion of segmental neuromeres into composite ganglia. Typically, the neuromeres of the palpal and ovigeral segments fuse into a bipartite SEG [59] and owing to its presence in the basally branching austrodecid genus *Pantopipetta* [46, 61], the bipartite SEG was previously traced to the last common ancestor of the pycnogonid crown group [61]. Our study reinforces this ground pattern by demonstrating a corresponding bipartite SEG in the genus *Austrodecus*, and thus a lack of intra-familial variability in the basally branching austrodecid lineage (Figs. 16A; 18B).

Further, the SEG of the two families Pycnogonidae and Phoxichilidiidae and of the ammotheid genus *Tanystylum* was previously known to include the leg 1 neuromere, resulting in an extended, tripartite composite ganglion [26, 43, 44, 46–48, 61, 69, 70, 72] (Figs. 16C, F; 17B). In this study, we reveal yet another case of a tripartite SEG in the hitherto understudied Rhynchothoracidae (Fig. 16B). So far, only a cursory description of the CNS in *Rhynchothorax mediterranus* was available, which highlights a similar forward shift of the VNC as in *R. australis*, but does not specifically address the question of an extended SEG [43]. Given the consistent conservation of SEG architecture within all other pycnogonid genera studied, we consider it highly likely that this trait can be extrapolated from *R. australis* to the monogeneric Rhynchothoracidae. Importantly, the tripartite SEG cannot be correlated to any striking structural features of the affiliated segments, such as the absence of the palps and/or ovigers. Rather, in the four taxa in question, these appendages are variably present and differently structured (Figs. 16B, C, F; 17B; see also [12]). In the case of the ovigers, their presence can even be sex-specific *within* species (in Pycnogonidae and Phoxichilidiidae, only the males possess ovigers) without affecting SEG composition. Conversely, other taxa with complete lack of palps and sex-specific absence of ovigers (Endeidae; Fig. 16E) retain a bipartite SEG and separate LG1. Due to this independence of SEG architecture and the suite and structure of affiliated appendages, the extended SEG qualifies as an internal anatomical character potentially useful for phylogenetic inference. Based on character mapping on the best supported pycnogonid phylogeny available, the tripartite SEG represents a derived character of Pycnogonidae and Rhynchothoracidae (Fig. 18B). This adds to other features (e.g., compact habitus; gonopores only on the ultimate leg pair; males carry a single egg package with both ovigers) previously suggested to support this clade [12, 43, 73]. Outside of this clade, a tripartite SEG was independently acquired two more times during pycnogonid evolution, in Phoxichilidiidae and in the ammotheid genus *Tanystylum* (Fig. 18B), concurrent with a previous interpretation [61].

### Fusion of other CNS ganglia

In addition to the bipartite SEG and LG1, two other cases of ganglion fusion were observed. The first relates to the brain (i.e., the posterior deutocerebrum affiliated with the cheliphores) and SEG (i.e., the anterior palpal neuromere) in *Callipallene* (Fig. 17F) and *Stylopallene*, two of the three callipallenid genera studied. As is the case for the tripartite SEG, this fusion is not correlated with striking changes in the external morphology: In spite of the widespread absence of palps also in other callipallenid genera, several representatives retain a distinct soma-free CEC, as here illustrated by *Parapallene* (Fig. 17G; see [59] for another example). Also in non-callipallenid families with cheliphores but without or with extremely reduced palps, a fusion of brain and SEG does not occur (Phoxichilidiidae: Fig. 16F; Pallenopsidae: Fig. 17C). Currently, the morphologically poorly defined Callipallenidae encompasses 17 genera [74] and taxonomic revisions are still ongoing [75–80]. Notably, however, the family is strongly indicated to represent a paraphyletic group in molecular phylogenetic studies [12, 19, 21] (Fig. 18). Given the high diversity of Callipallenidae and the underrepresentation of the various genera in phylogenetic analyses and in this study, it is at this stage premature to assess whether the fusion of brain and SEG may qualify as a derived character of a monophyletic subset in the callipallenid grade.

The second instance of a fusion occurs between LG3 and LG4 of *R. australis* (Rhynchothoracidae) (Fig. 16B). Remarkably, the extent to which both ganglia are fused was found to vary across specimens and therefore cannot serve as a reliable character for outgroup comparison. To our knowledge, the only previous account of an intra-specifically variable fusion relates to LG3 and LG4 in *P. litorale* (Pycnogonidae), which Winter [26] reported to be merged in many, but not all specimens studied. As monophyletic Pycnogonidae + Rhynchothoracidae is supported in molecular phylogenetic analyses [12, 19] (Fig. 18) and by several morphological and anatomical features (see previous discussion section), this phenomenon may point to a progressing evolutionary trend of VNC fusion in this clade. Speaking against this notion, however, is the fact that none of the studies prior or subsequent to Winter’s account found any evidence for a fusion of LG3 and LG4 in normally developed *P. litorale* [44, 60, 61, 69] (Fig. 16C), leading to the conclusion that he likely misinterpreted his data.

### Absence of ISNs correlates with trunk segment fusion and longitudinal muscle reduction

The pycnogonid ISNs between the four leg ganglia were first described in one of the earliest anatomical studies [44], but with few exceptions [48, 59] found no further mention in subsequent works dealing with the VNC (e.g., [26, 46, 47]). Here, we have shown that the ISNs serve the longitudinal segmental trunk musculature (especially the ventral part). Identification of longitudinal musculature as main innervation target of the ISNs is also indirectly supported by the strong correlation between trunk segment fusion and its concurrent reduction of longitudinal musculature on the one hand, and the accompanying lack of the respective ISNs on the other. Based on this pronounced interdependency with external morphology, the ISN patterns are resolved as an anatomical character contributing no additional phylogenetic information. Beyond that, even the external fusion of trunk segments is of limited value for phylogenetic inference at the family level, as it occurs within well-supported families. For instance, while the colossendeid genus *Rhopalorhynchus* displays a fully segmented trunk [81], the genus *Colossendeis* as well as the polymerous *Decolopoda* and *Dodecolopoda* feature fused trunk segments [15, 82]. The same may hold for the Ascorhynchidae, if the recently proposed inclusion of the genus *Paranymphon* finds further corroboration in future studies [12]. On top of that, varying trunk fusion patterns are found even within single genera, such as *Achelia*, *Ammothella* and *Anoplodactylus* [15]. As a consequence, also the linked changes in the ISN patterns show a disjunct distribution when mapped upon the pycnogonid phylogeny (Fig. 18C).

Regardless of this, the presence of paired ISNs between free trunk segments can be securely traced to the last common ancestor of the pycnogonid crown group (Fig. 18C). Owing to the interdependency of ISNs and free trunk segments in extant sea spiders, these nerves may be even tentatively extrapolated to stem lineage representatives with free trunk segments [83, 84]. Also in mandibulate taxa, the VNC features paired ISNs [40, 58, 85–88], which are known to encompass motoneuron projections targeting body wall muscles in several representatives [89–92]. As Pycnogonida represent the earliest diverging lineage in the chelicerate crown group [7, 9], the presence of paired ISNs thus reinforces their reconstruction in the VNC of the last common ancestor of Arthropoda (e.g., [54]).

### Multi-branching midgut diverticula evolve in the family Pycnogonidae

One of the most conspicuous anatomical features of Pycnogonida is the presence of diverticula that extend from the central midgut tube into the legs and variably also into the cheliphore (if present) or the proboscis [3, 49]. While periodic contractions of the midgut and its diverticula have been noticed early on [43, 63], this gut peristalsis was only recently demonstrated to be crucial for oxygen transport, as it actively supports the relatively weakly developed tube-shaped dorsal heart in hemolymph circulation in the long appendages [64]. Almost without exception, the leg diverticula represent simple linear extensions into the legs, which unequivocally qualifies as the ancestral condition of the pycnogonid crown group (Fig. 18D). Beyond variations in the form of A–P shifts of the diverticula’s divergence points from the central tube and their penetration depth into the legs (Figs. 16, 17), the basic layout experiences a more striking structural change in the family Pycnogonidae. In *Pycnogonum litorale*, the leg diverticula bifurcate repeatedly to form a multi-branching network in the legs (Fig. 16C). This confirms previous reports on the same species [44, 68, 69], but we here added two additional congeners in our analyses to assess the intra-generic stability of this pattern. Notably, the midgut of the largest *P. diceros* displays an even more intricate branching pattern that even includes additional dorsal projections from the central tube. By contrast, the likewise large *P. gaini* possesses simple, linear leg diverticula, which aligns with similar reports on two smaller Mediterranean *Pycnogonum* species [43]. Based on outgroup comparison, this indicates that the unique multi-branching diverticula evolved within Pycnogonidae (Fig. 18D). However, as a stable phylogeny for the family is currently missing, this conclusion remains preliminary and may have to be reevaluated as more data become available.

Outside of Pycnogonidae, deviations from linear leg diverticula have been reported for the two endeid species *E. nodosa* Hilton, 1942 and *E. flaccida* Calman, 1923. Here, small pouches protrude at irregular intervals along the entire length of each diverticulum, giving it an uneven outline [93, 94]. Proximal bifurcations that lead to multiple longer branches extending for some distance in parallel through the legs are, however, absent. As these irregular pouches are lacking in *E. spinosa* studied here (Fig. 16E), they likely evolved within Endeidae (Fig. 18D).

### Is the evolution of multi-branching leg diverticula linked to heart reduction?

Of all pycnogonids studied with regard to the circulatory system [43, 46, 65–67], *P. litorale* and *P. diceros* are the only two species known to lack a heart [65, 69]. This is surprising, given that respiration in pycnogonids is reliant on oxygen diffusion through the cuticle and pores therein [95] and that especially a more compact habitus as is characteristic of Pycnogonidae seems to call for active oxygen transport to centrally located tissues. In the light of this, the more intricate structure of the gut diverticula may serve to compensate for the lack of a heart by guaranteeing sufficient hemolymph propulsion via midgut peristalsis alone. This ad hoc functional explanation is obviously challenged by the simple midgut layout in *P. gaini* (which is larger than *P. litorale*), coupled to the fact that we found no convincing evidence for a heart in the µCT scan of this species either (not shown). To reliably evaluate to what extent the evolution of a more elaborate midgut branching pattern coheres with heart reduction, future phylogenetic studies should therefore aim to resolve in-group relationships of Pycnogonidae in more detail, accompanied by dedicated internal anatomical investigation of all species analyzed.

## Conclusions

In this study, we applied non-invasive µCT and 3D reconstruction to gain directly comparable in-situ representations of the CNS and midgut layout across all pycnogonid families. Against the backbone of a recently published stable pycnogonid phylogeny [12], this for the first time enables to reliably elucidate evolutionary trends in both organ systems, and to disentangle independently evolved versus potentially apomorphic characters at the gross anatomical level accessible with the approach. Similar to the notoriously homoplastic external morphology of Pycnogonida (especially in the head region), the gross CNS layout displays several independently evolved traits in phylogenetically distant families. This includes a variable A–P shift of VNC ganglia, which is to some extent correlated with multiple cases of trunk elongation/compaction, or the loss of ISNs in concert with trunk segment fusion. In contrast to this, other characters help to underpin close phylogenetic affinities in sub-branches of the tree supported by molecular studies, as exemplified by the tripartite SEG in Pycnogonidae and the enigmatic Rhynchothoracidae. To leverage the full potential of pycnogonid neuroanatomy for phylogenetic interrogation, future studies should now seek to decipher brain and ventral ganglion neuroarchitecture at higher resolution. In this regard, pioneering work on specific neuronal subsets already revealed promising character sets with unequivocal homologies in addition to inter-familial differences [60], in correspondence to similar studies in other arthropod groups (e.g., [35, 96, 97]).

## Methods

### Species sampling and fixation

Specimens were collected in various locations (1) during field trips, (2) at marine research stations, such as Station Biologique de Roscoff (Bretagne, France) and Rothera Research Station (British Antarctic Survey, Antarctica), as well as (3) during cruises of research vessels, such as RV Polarstern [98]. The main priority for species selection was the coverage of all major pycnogonid lineages (families). In order to evaluate the intra-familial stability of CNS and midgut patterns in morphologically highly diverse families (e.g., Ammotheidae, Callipallenidae), representatives of different genera with deviating habitus were included. To further assess whether body size impacts internal anatomical patterns, differently sized congeners were exemplarily examined (e.g., genus *Pycnogonum*).

Sample availability for more elusive families (e.g., Austrodecidae, Rhynchothoracidae, Colossendeidae) was restricted to material collected and preserved during previous research cruises with their dedicated fixation protocols (e.g., long-term storage in borax-buffered 10% formaline in sea water; 70% or 96% ethanol). In these cases, targeted use of fixatives shown to be beneficial for soft tissue contrast in µCT studies, such as Bouin’s fluid (10% formaldehyde, 5% glacial acetic acid in saturated aqueous picric acid) [99], or immunohistochemical labeling (e.g., 4% paraformaldehyde [PFA] for few hours only) was not possible. All species studied, collection sites, fixatives and methods used, and number of specimens investigated are listed in Additional file 1: Table S1.

### Micro-computed X-ray tomography (µCT)

Specimens were either fixed and stored in (1) Bouin’s fluid, (2) borax-buffered 10% formaline in sea water, (3) 70% ethanol or (4) 96% ethanol. Bouin- and formaline-stored samples were briefly rinsed in phosphate-buffered saline (PBS; 1.86 mM NaH_2_PO_4_, 8.41 mM Na_2_HPO_4_, 175 mM NaCl, pH 7.4), transferred into deionized water and dehydrated in an ascending ethanol series. For specimens already preserved in ethanol, the ascending ethanol series was continued up to 99.5%. Samples were subsequently incubated in a solution of 2% iodine (resublimated; Carl Roth; #X864.1) in 99.5% ethanol for 48–72 h at room temperature (RT). After rinsing in 99.5% ethanol (3–4 × 10 min), they were either directly scanned in ethanol-filled plastic tubes (wet scan) or critical point-dried using a Leica EM CPD300 (dry scan). Dried specimens were placed in plastic tubes for overview scans and subsequently glued to plastic welding rods for trunk scans at a higher resolution. Data acquisition was performed with an Xradia MicroXCT-200 (Carl Zeiss Microscopy) using the XMController software (Carl Zeiss Microscopy). Scans were run under 40 kV/200 µA/8 W or 30 kV/200 µA/6 W settings. The objective used (0.39×, 4×, 10×, 20×) and exposure times (ranging from 0.75 to 7 s) were individually adjusted, depending on specimen size and tissue contrast. To improve the signal-to-noise ratio, binning 2 was applied during data acquisition. Tomography projections were reconstructed with full resolution (binning = 1) using the XMReconstructor software (Carl Zeiss Microscopy) with TIFF format image stacks as output. For several species with elongated trunk, serial scans along the A–P axis were conducted and merged in the XMController software (plugin_stitch.dll) according to the manufacturer’s manual. Especially samples that were long term-stored in formaline showed comparably low tissue contrast.

### Specimen fixation and dissection for immunohistochemistry

Most specimens were fixed overnight at 4 °C in 4% paraformaldehyde in sea water (PFA/SW; 16% methanol-free formaldehyde [Electron Microscopy Sciences, #15710] diluted 1:4 in 0.2 µm pore-filtered natural sea water), rinsed in PBS and either directly processed or transferred to cryoprotectant buffer (0.5 × PBS, 300 g/L sucrose, 30% (v/v) ethylene glycol, 10 g/L polyvinylpyrrolidone) for long-term storage at -20 °C. For some species, only specimens stored for several years in formaline fixative were available (see Additional file 1: Table S1). They were rinsed in PBS for several days prior to further processing. For the study of CNS whole mounts, the CNS was manually dissected in PBS under a stereomicroscope.

### Immunohistochemistry, mounting of samples and data acquisition

In some species, the CNS features a prominently developed neural sheath surrounded by connective tissue (e.g., *Pycnogonum litorale*). To facilitate antibody penetration, the CNS was pre-treated with Proteinase K (1 × solution, Thermo Fisher Scientific, #C10617) in PBS for 15 min, rinsed in several changes of PBS and post-fixed in 4% PFA/PBS for 15 min at RT. Alternatively, samples with prominent sheath were exposed to a mixture of collagenase and hyaluronidase (Sigma-Aldrich, #C0130 and #H3884, respectively; each at 1 mg/ml in PBS) for 1 h at 37 °C. As first tests showed that this pre-treatment negatively impacts immunolabeling for synapsin, it was exclusively used for tubulin immunolabeling.

Dissected CNSs were permeabilized for ≥ 2 h in several changes of PBTx (PBS + 0.5% Triton-X + 1.5% dimethyl sulfoxide) at RT. Prior to incubation in primary and secondary antibodies/-sera, samples were blocked in PBTx + 5% normal goat serum (Thermo Fisher Scientific, #31,873) for at least 1 h at RT. All primary and secondary antibodies/-sera (Table 2) were diluted in PBTx; incubation times lasted 72–120 h and were followed by rinsing in PBTx with gentle rotation for at least 6 h at RT, with occasional extension overnight at 4 °C. Omission of primary antibodies/-sera in the procedure resulted in absence of signal. The nucleic acid marker Hoechst 33,342 (Thermo Fisher Scientific, #H1399, 1 µg/mL in PBS) was used to stain cell nuclei. Incubation lasted minimally 1 h at RT and was occasionally extended overnight at 4 °C. Stained samples were transferred into non-hardening Vectashield® Mounting Medium (Vector Laboratories, Inc. #H-1000, RRID:AB_2336789) and placed on microscopic slides. To avoid squeezing, small pieces of Surgident periphery wax were placed under the cover slip corners.

**Table 2:** Antibodies/antisera, research resource identifiers and dilutions

Antibody/-serum	#RRID	Dilution
Primary		
Mouse anti-SYNORF1 (*Drosophila* synapsin-1), monoclonal, supernatant, Developmental Studies Hybridoma Bank, #3C11	AB_528479	1:100
Mouse anti-acetylated tubulin IgG 2b isotype, clone 6-11 B-1, Sigma-Aldrich, #T6793	AB_477585	1:200–300
Rat anti-alpha tubulin IgG2a isotype, clone YL1/2, Thermo Fisher Scientific, #MA1-80017	AB_2210201	1:500
Secondary		
Cy3-AffiniPure goat anti-mouse IgG (H + L), polyclonal, Jackson ImmunoResearch Labs, #115-165-166	AB_2338692	1:250
Alexa Fluor647-AffiniPure goat anti-rat IgG (H + L), polyclonal Jackson ImmunoResearch Labs, #112-605-167	AB_2338404	1:250

Confocal laser scanning microscopy (CLSM) was performed with a Leica DMI 6000 CS microscope with a Leica TCS SP5 II scan unit (RRID:SCR_018714). Laser lines were chosen according to fluorochrome excitation spectra (405 nm for Hoechst; 543 nm for Cy3; 633 nm for Alexa Fluor 647). The Z-increment between optical planes ranged from 0.5 to 2 µm, depending on objective used (10x, 20x, 63x) and required resolution. On average, samples long term-fixed in formaline displayed less homogeneous tissue labeling, especially in the case of the tubulin antibodies used.

### Specification of primary antibodies/-sera

For full details of primary and secondary antibodies/-sera and their dilutions see Table 2.

The monoclonal mouse anti-SYNORF1 antibody (deposited at Developmental Studies Hybridoma Bank by E. Buchner, University Hospital Würzburg) was raised against a *Drosophila melanogaster* GST-synapsin fusion protein and has proved a useful marker for synaptic neuropil in many arthropod taxa, including chelicerates [58, 100–104].

To visualize cytoskeletal microtubules, a monoclonal mouse antibody raised against acetylated alpha-tubulin from the sea urchin *Strongylocentrotus purpuratus* was used. Acetylation is a post-translational modification of alpha-tubulin in which an acetyl group is reversibly added to Lys^40^ by a specific acetylase. It is found in many cell types and is often encountered in stable microtubule assemblies [105]. The suitability of this antibody to reveal the microtubule-rich neurite bundles in the nerves and tracts has been demonstrated for a range of arthropod taxa [92, 106–108], including pycnogonids [10, 60, 61].

For simultaneous labeling of tubulin-rich structures and synaptic neuropil, a monoclonal rat antibody raised against the tyrosinated alpha-subunit of yeast tubulin was applied. Tyrosinated alpha-tubulin represents the gene-encoded protein of alpha-tubulin, which is characterized by a C-terminal tyrosine that can be post-translationally removed via detyrosination [104]. According to the manufacturer’s specifications, the antibody labels tyrosinated alpha-tubulin in a wide range of metazoan taxa, and its suitability for neuroanatomical studies in pycnogonids has been recently demonstrated [109].

### Data analysis, visualization and presentation

The µCT image stacks were analyzed and processed with the software package Amira (version 5.6; FEI Visualization Sciences Group, RRID:SCR_007353) in similar fashion as previously described [110]. In brief, the CNS and midgut were manually segmented as different materials in one label field. By applying 3D volume rendering (module “Volren”, mode “VRT”) with user-defined shading, the two organ systems were visualized. Color and transparency of the 3D volume renderings were defined by adjusting the color field and alpha-scale values of the transfer functions for each material. The 3D volume renderings of the midgut were additionally combined with 3D surfaces generated from the segmented materials to enhance the distinction of midgut diverticula and their branching patterns. As a positional reference system, the unsegmented structures of the data stack were included in semi-transparent grayscale values. To improve visibility of the CNS and midgut further, filtered oblique slicers were applied to remove concealing grayscale structures in the different perspectives. Separate labeling of the head appendage and leg nerves of the right body half and their subsequent assignment to a separate material enabled transient removal of these structures to improve clarity in oblique views of the CNS.

The obtained CLSM image stacks were processed with Imaris (ver. 7.00; Bitplane AG, Zurich, Switzerland, RRID:SCR_007370). Software tools were applied as previously described [38, 111], including manual segmentation and the use of optical clipping planes to remove non-target structures.

Global contrast, brightness and sharpness of snapshots were adjusted using Adobe Photoshop (ver. 12.1, Adobe Systems Incorporated, San Jose, CA, USA, RRID:SCR_014199). Black-and-white inversion was applied to some images to improve visibility of delicate nerves. Figures were assembled with Adobe Illustrator (ver. 15.1, Adobe Systems Incorporated, RRID:SCR_010279).

### Terminology

Neuroanatomical terminology follows [112] where applicable. Species names are updated according to [74].

## Supplementary Information

**Additional file 1: Table S1.** List of species studied, collection sites and methods applied.

**Additional file 2: Figure S1.** The subesophageal and leg 1 ganglia in various pycnogonid families. Immunolabeled samples, CLSM scans (MIP). The black arrows indicate borders between ganglia (**A-C**) and longitudinal connectives (**H**, **I**). The small white arrowheads point to the roots of the paired ventral proboscis nerve. Stars (**E**, **F**) mark areas in which nerve roots have been damaged during CNS dissection. **A-G:** Synapsin (red) and tyrosinated alpha-tubulin (white) labeling. **A-C:** Representatives with directly adjoining but anatomically separate subesophageal and leg 1 ganglia. Note distinct separation of the ganglionic neuropil cores. **A:** *Nymphon gracile* (Nymphonidae), subadult. **B:** *Austrodecus glaciale* (Austrodecidae), adult female. **C:** *Achelia echinata* (Ammotheidae), adult male. **D-G:** Representatives with extended subesophageal ganglion. Note fusion of the neuropil cores of the palpal, ovigeral and leg 1 neuromeres. **D:** *Rhynchothorax australis* (Rhynchothoracidae), adult male. **E:** *Tanystylum orbiculare* (Ammotheidae), adult male. **F:** *Pycnogonum litorale* (Pycnogonidae), subadult male. **G:** *Phoxichilidium femoratum* (Phoxichilidiidae), adult female. **H-J:** Acetylated alpha-tubulin labeling (white) with nuclear counterstain (blue). **H, I:** Representatives with widely separated subesophageal and leg 1 ganglia interconnected by long connectives. **H:** *Ascorhynchus auchenicus* (Ascorhynchidae), subadult. **I:** *Nymphonella tapetis* (Ascorhynchidae), adult male. **J:** *Anoplodactylus pygmaeus* (Phoxichilidiidae), adult male. Note the extended subesophageal ganglion as in other phoxichilidiids (**G**).

**Additional file 3: Figure S2.** The central nervous system and midgut in the trunk of *Pentanymphon antarcticum* (Nymphonidae). Reconstructions of the CNS (3D volume rendering, green) and midgut (3D surface, magenta) based on a µCT scan of an adult male. The white arrowhead points to the posteriorly projecting proctodeal nerve. For better pattern visualization, all major nerves of the right body half were virtually removed in **A** (right side) and **B**. **A:** Oblique antero-lateral view. Right top corner: overview of the complete specimen. Left side: complete CNS and midgut reconstruction. Right bottom corner: anterior portion of the CNS and eyes. **B:** Lateral view of the CNS with and without the midgut structures (top and bottom, respectively). For reference, unreconstructed parts of the right body half are shown in transparent gray. **C:** Ventral view. Left side: CNS in grayscale. The black arrowheads point to the intersegmental nerves. Right side: CNS and midgut. For reference, unreconstructed dorsal parts of the trunk are shown in transparent gray.

**Additional file 4: Figure S3.** Intersegmental nerves in the VNC of various pycnogonid families. CLSM scans of posterior VNC ganglia, acetylated alpha-tubulin immunolabeling (**A**–**G**) or synapsin (red) and tyrosinated alpha-tubulin (white) double immunolabeling (**F**). The large black arrowheads point to the intersegmental nerves. Small black arrowheads indicate the branching point of the intersegmental nerves from the connectives. The asterisks (**B**, **F**, **G**) highlight a gap between antero-posteriorly adjoining ganglia. Black stars (**A**–**F**) mark posterior commissure(s) in leg ganglion 4, indicative of the fusion of transient posterior ganglion anlagen. **A:** *Callipallene brevirostris* (Callipallenidae). Note the extremely delicate ultimate intersegmental nerve (small arrowheads). **B:** *Stylopallene cheilorhynchus* (Callipallenidae). The penultimate intersegmental nerve emerges antero-laterally from the soma cortex of leg ganglion 3. Arrows point to connective tissue attaching to the connectives. **C:** *Phoxichilidium femoratum* (Phoxichilidiidae). **D:** *Achelia echinata* (Ammotheidae). Note the lack of an intersegmental nerve between leg ganglia 3 and 4. **E:** *Tanystylum orbiculare* (Ammotheidae). Note the complete absence of intersegmental nerves. **F:** *Austrodecus glaciale* (Austrodecidae). Note the protruding vestigial posterior ganglion anlage. **G**, **H:** *Rhynchothorax australis* (Rhynchothoracidae). Note the anatomical separation of leg ganglia 3 and 4 (**G**) versus their fusion (**H**). Small black arrowheads (**G**) trace the ultimate intersegmental nerve from the connective through the soma cortex of leg ganglion 4 (shown in blue autofluorescence signal). The white arrowheads (**H**) indicate the adjoining neuropil cores of the leg 3 and 4 neuromeres.

**Additional file 5: Figure S4.** Arrangement of brain and ventral ganglia in various pycnogonid families. Optical sagittal sections; µCT scans. Where discernible, the white arrowheads point to the vestigial posterior ganglion anlagen at the dorso-posterior side of leg ganglion 4. **A:** *Callipallene brevirostris* (Callipallenidae), male. **B:** *Ascorhynchus castellioides* (Ascorhynchidae), male. **C:** *Pallenopsis vanhoeffeni* (Pallenopsidae), subadult. **D:** *Ammothea clausi* (Ammotheidae), male. **E:** *Ammothea* sp. (Ammotheidae), female. **F:** *Ammothella biunguiculata* (Ammotheidae), male. **G:** *Ammothella longipes* (Ammotheidae), female.

**Additional file 6: Figure S5.** The central nervous system and midgut in the trunk of *Stylopallene cheilorhynchus* (Callipallenidae). Reconstructions of the CNS (3D volume rendering, green) and midgut (3D surface, magenta) based on a µCT scan of an adult male. The white arrowheads point to the origin of the posteriorly projecting proctodeal nerve. Asterisks indicate incompletely reconstructed portions of the optic nerve (insufficient resolution and tissue damage). For better pattern visualization, all major nerves of the right body half were virtually removed in **A** (right side) and **C**. **A:** Oblique antero-lateral view. Left bottom corner: overview of the complete specimen. Top: complete CNS and midgut reconstruction. Right bottom corner: anterior portion of the CNS and eyes. **B:** Ventral view. Left side: CNS in grayscale. The black arrowhead points to an intersegmental nerve. Right side: CNS and midgut. For reference, unreconstructed dorsal parts of the trunk are shown in transparent gray. **C:** Lateral view of the CNS with and without the midgut structures (left and right, respectively). The white arrow highlights the fusion area of brain and subesophageal ganglion. For further reference, unreconstructed parts of the right body half are shown in transparent gray. Double arrows indicate dorsal longitudinal musculature in the cephalosoma and trunk segment 2. In the fused trunk segments 3 and 4, longitudinal musculature is missing (stippled oval).

**Additional file 7: Figure S6**. Comparison of the midgut branching pattern in the trunk of three species of the family Pycnogonidae. **A:** Volume renderings of µCT overview scans of complete specimens (from top to bottom: *Pycnogonum litorale*, *P. gaini*, *P. diceros*), dorsal view. Specimens are up to scale to illustrate the size differences. **B**–**D’:** 3D surfaces of CNS (green) and midgut (purple) reconstructions of the three species studied. For each species, an oblique antero-lateral view (left column) and a dorsal view (right column) is shown. **B, B’:** *P. litorale*. Note the widely spaced divergence points of the leg diverticula along the central midgut tube and their proximal bifurcation and further branching. **C, C’:** *P. gaini*. Note shorter distance between the leg diverticula’s divergence points and their simple linear structure. **D, D’:** *P. diceros.* Note closely spaced divergence points of the leg diverticula and their complex pattern of several sub-branches, including a dorsal projection that extends medially into the dorsal trunk (double arrowheads in **D**). Unpaired projections of the midgut extend into the dorso-median tubercles of the anterior trunk segments (arrows in **D**). Between the central gut tube and the right leg diverticulum 4, a non-bilaterally symmetrical secondary connection is present (arrowhead in **D’**).

**Additional file 8: Figure S7.** The central nervous system and midgut in the trunk of *Pycnogonum diceros* (Pycnogonidae). Reconstructions of the CNS (3D volume rendering, green) and midgut (3D surface, magenta) based on a µCT scan of an adult female. The white arrowheads point to the origin of the posteriorly projecting proctodeal nerve. White arrows indicate midgut projections into the dorso-median tubercles of the cephalosoma and of trunk segment 2. For better pattern visualization, all major nerves of the right body half were virtually removed in **A** (right side) and **B**. **A:** Oblique antero-lateral view. Right top corner: overview of the specimen. Left side: complete CNS and midgut reconstruction. The double arrowheads indicate projections from each leg’s dorsal diverticulum branch that extend dorso-medially into the trunk. Note that the voluminous proboscis diverticulum covers the view on the brain almost completely. Right bottom corner: anterior portion of the CNS. The eyes are not shown (not included in the µCT scan). **B:** Lateral view of the CNS with and without the midgut structures (top and bottom, respectively). The dorso-distal portions of the leg diverticula are highlighted (stippled lines) to illustrate their extensive branching in the trunk. For reference, unreconstructed parts of the right body half are shown in transparent gray. **C:** Ventral view. Left side: CNS in grayscale. Black arrows point to an additional branch of the leg nerves that extends to the body wall. Right side: CNS and midgut. For reference, unreconstructed dorsal parts of the trunk are shown in transparent gray.

**Additional file 9: Figure S8**. The central nervous system and midgut in the trunk of *Pantopipetta armoricana* (Austrodecidae). Reconstructions of the CNS (3D volume rendering, green) and midgut (3D surface, magenta) based on a µCT scan of a subadult. The white arrowheads indicate the origin of the posteriorly projecting proctodeal nerve. The white arrows indicate a small anterior midgut protrusion. Note that the eyes are not included in the scan, owing to the extreme elongation of the ocular tubercle. For better pattern visualization, all major nerves of the right body half were virtually removed in **A** (right side) and **B**. **A:** Oblique antero-lateral view. Right top corner: overview of the specimen. Left side: complete CNS and midgut reconstruction. Right bottom corner: anterior portion of the CNS and eyes. **B:** Lateral view of the CNS with and without the midgut structures (top and bottom, respectively). For reference, unreconstructed parts of the right body half are shown in transparent gray. **C:** Ventral view. Left side: CNS in grayscale. The black arrowheads point to the intersegmental nerves. Right side: CNS and midgut. For reference, unreconstructed dorsal parts of the trunk are shown in transparent gray.

## Data Availability

The data used and analyzed during the study are included in this published article and its supplementary information files. Raw datasets are available from the corresponding author upon reasonable request.
