# Supplementary figures and images for: A microCT-based atlas of the central nervous system and midgut in sea spiders (Pycnogonida) sheds first light on evolutionary trends at the family level
Source: Front Zool. 2022 Mar 31;19:14. doi: 10.1186/s12983-022-00459-8 (PMC8973786; doi:10.1186/s12983-022-00459-8)

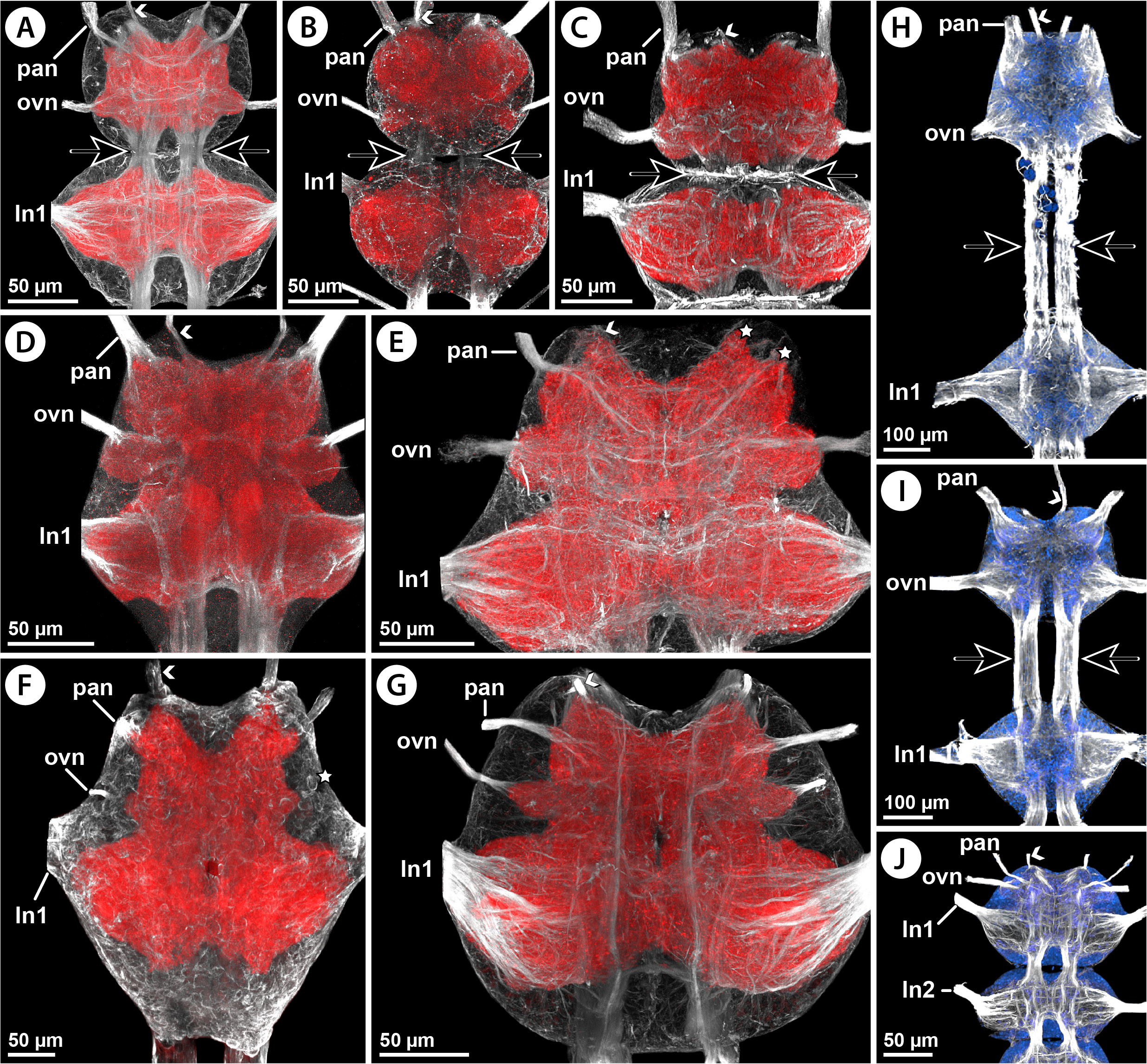

Supplement: Supplementary file 2 — Additional file 2: Figure S1. The subesophageal and leg 1 ganglia in various pycnogonid families. Immunolabeled samples, CLSM scans (MIP). The black arrows indicate borders between ganglia (A-C) and longitudinal connectives (H, I). The small white arrowheads point to the roots of the paired ventral proboscis nerve. Stars (E, F) mark areas in which nerve roots have been damaged during CNS dissection. A-G: Synapsin (red) and tyrosinated alpha-tubulin (white) labeling. A-C: Representatives with directly adjoining but anatomically separate subesophageal and leg 1 ganglia. Note distinct separation of the ganglionic neuropil cores. A: Nymphon gracile (Nymphonidae), subadult. B: Austrodecus glaciale (Austrodecidae), adult female. C: Achelia echinata (Ammotheidae), adult male. D-G: Representatives with extended subesophageal ganglion. Note fusion of the neuropil cores of the palpal, ovigeral and leg 1 neuromeres. D: Rhynchothorax australis (Rhynchothoracidae), adult male. E: Tanystylum orbiculare (Ammotheidae), adult male. F: Pycnogonum litorale (Pycnogonidae), subadult male. G: Phoxichilidium femoratum (Phoxichilidiidae), adult female. H-J: Acetylated alpha-tubulin labeling (white) with nuclear counterstain (blue). H, I: Representatives with widely separated subesophageal and leg 1 ganglia interconnected by long connectives. H: Ascorhynchus auchenicus (Ascorhynchidae), subadult. I: Nymphonella tapetis (Ascorhynchidae), adult male. J: Anoplodactylus pygmaeus (Phoxichilidiidae), adult male. Note the extended subesophageal ganglion as in other phoxichilidiids (G). [file 12983_2022_459_MOESM2_ESM.tif]

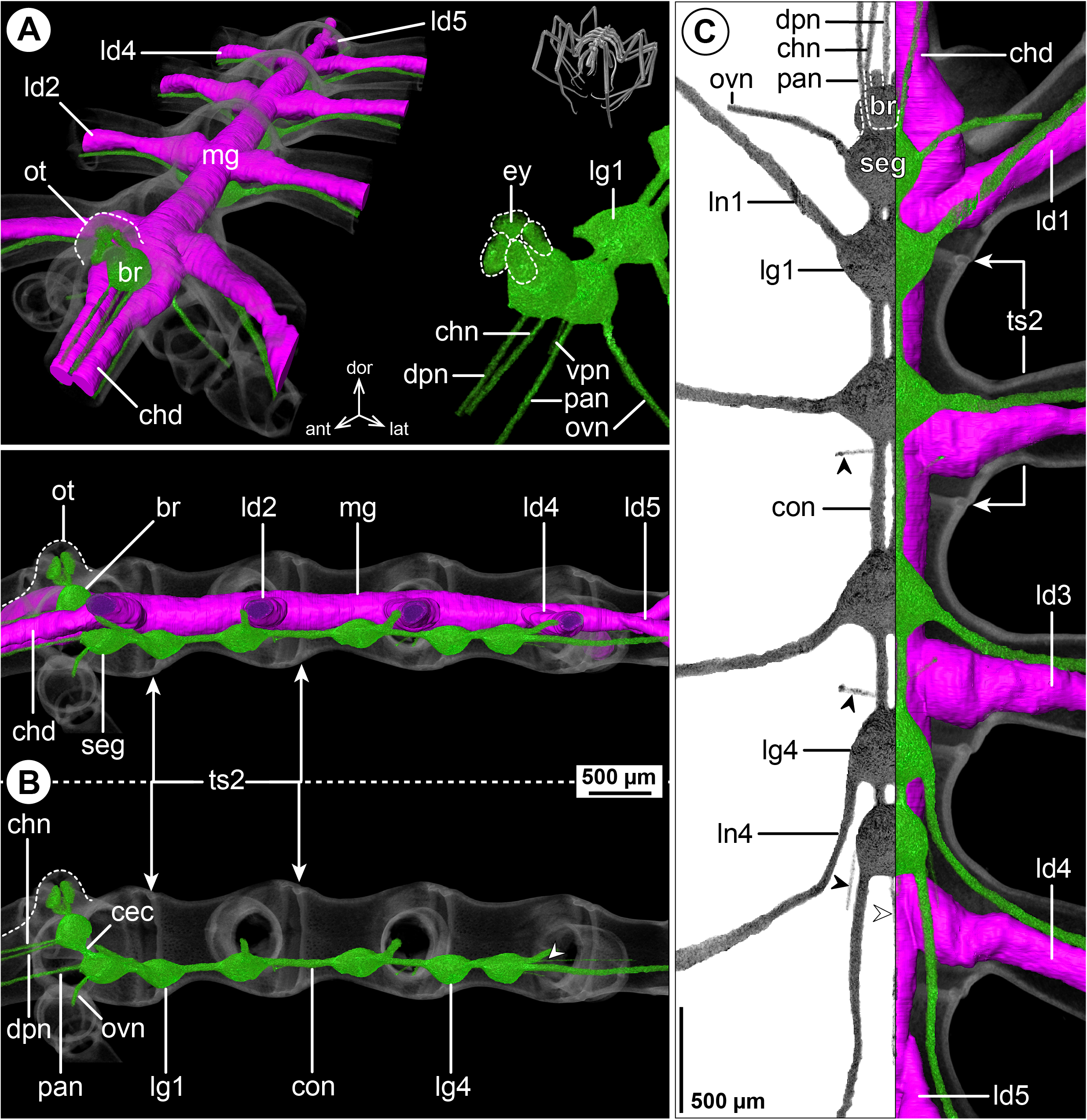

Supplement: Supplementary file 3 — Additional file 3: Figure S2. The central nervous system and midgut in the trunk of Pentanymphon antarcticum (Nymphonidae). Reconstructions of the CNS (3D volume rendering, green) and midgut (3D surface, magenta) based on a µCT scan of an adult male. The white arrowhead points to the posteriorly projecting proctodeal nerve. For better pattern visualization, all major nerves of the right body half were virtually removed in A (right side) and B. A: Oblique antero-lateral view. Right top corner: overview of the complete specimen. Left side: complete CNS and midgut reconstruction. Right bottom corner: anterior portion of the CNS and eyes. B: Lateral view of the CNS with and without the midgut structures (top and bottom, respectively). For reference, unreconstructed parts of the right body half are shown in transparent gray. C: Ventral view. Left side: CNS in grayscale. The black arrowheads point to the intersegmental nerves. Right side: CNS and midgut. For reference, unreconstructed dorsal parts of the trunk are shown in transparent gray. [file 12983_2022_459_MOESM3_ESM.tif]

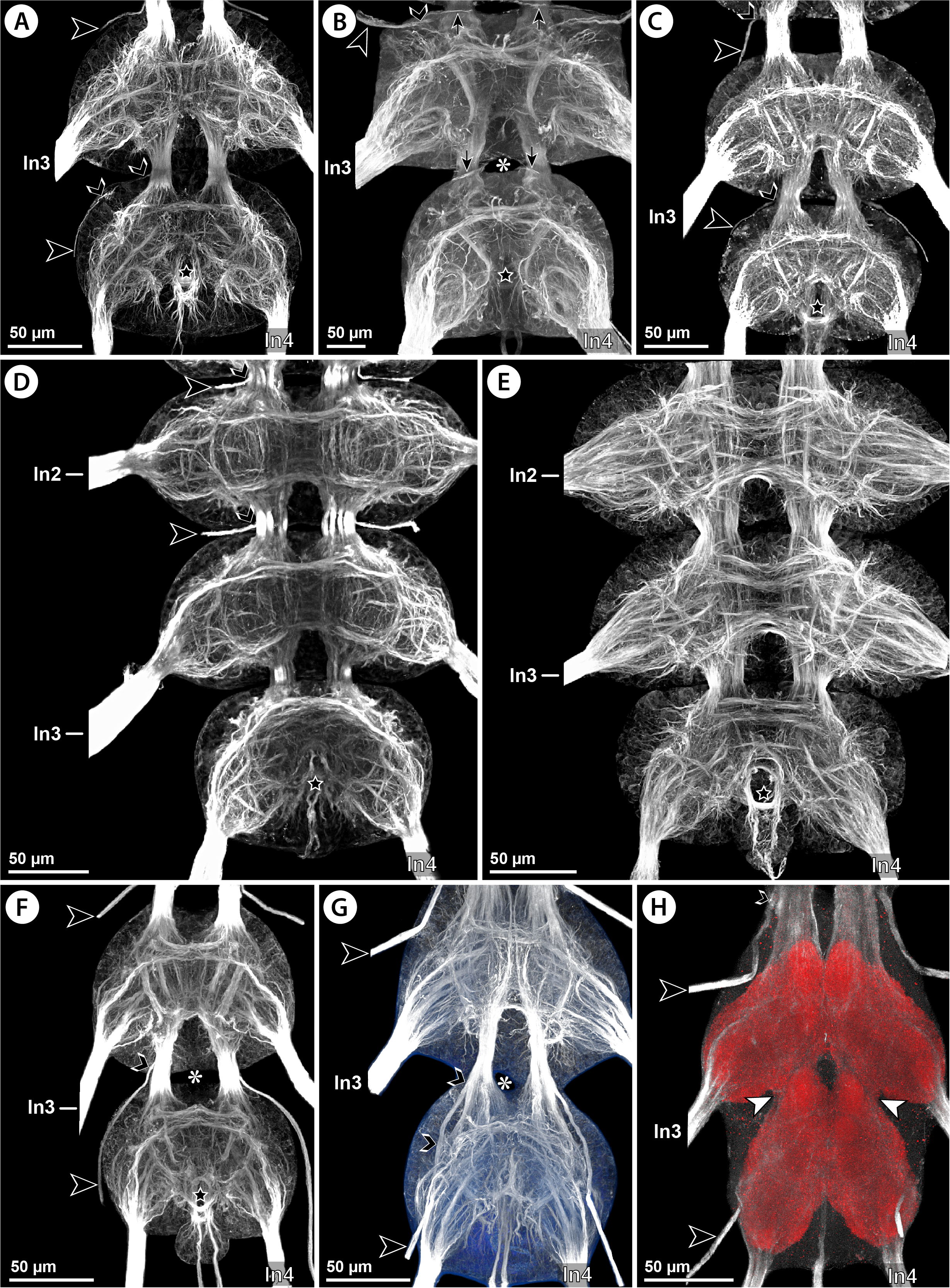

Supplement: Supplementary file 4 — Additional file 4: Figure S3. Intersegmental nerves in the VNC of various pycnogonid families. CLSM scans of posterior VNC ganglia, acetylated alpha-tubulin immunolabeling (A–G) or synapsin (red) and tyrosinated alpha-tubulin (white) double immunolabeling (F). The large black arrowheads point to the intersegmental nerves. Small black arrowheads indicate the branching point of the intersegmental nerves from the connectives. The asterisks (B, F, G) highlight a gap between antero-posteriorly adjoining ganglia. Black stars (A–F) mark posterior commissure(s) in leg ganglion 4, indicative of the fusion of transient posterior ganglion anlagen. A: Callipallene brevirostris (Callipallenidae). Note the extremely delicate ultimate intersegmental nerve (small arrowheads). B: Stylopallene cheilorhynchus (Callipallenidae). The penultimate intersegmental nerve emerges antero-laterally from the soma cortex of leg ganglion 3. Arrows point to connective tissue attaching to the connectives. C: Phoxichilidium femoratum (Phoxichilidiidae). D: Achelia echinata (Ammotheidae). Note the lack of an intersegmental nerve between leg ganglia 3 and 4. E: Tanystylum orbiculare (Ammotheidae). Note the complete absence of intersegmental nerves. F: Austrodecus glaciale (Austrodecidae). Note the protruding vestigial posterior ganglion anlage. G, H: Rhynchothorax australis (Rhynchothoracidae). Note the anatomical separation of leg ganglia 3 and 4 (G) versus their fusion (H). Small black arrowheads (G) trace the ultimate intersegmental nerve from the connective through the soma cortex of leg ganglion 4 (shown in blue autofluorescence signal). The white arrowheads (H) indicate the adjoining neuropil cores of the leg 3 and 4 neuromeres. [file 12983_2022_459_MOESM4_ESM.tif]

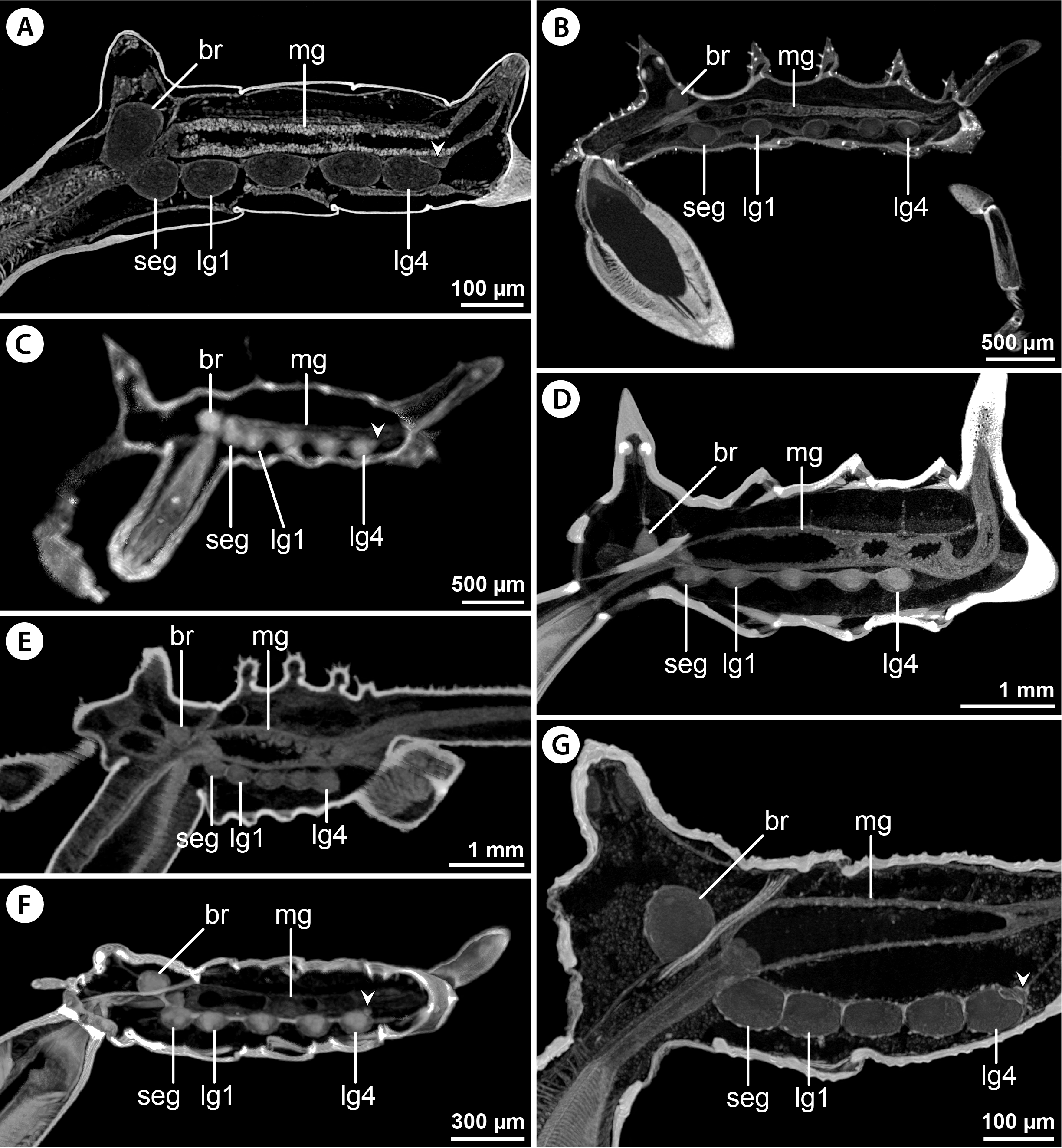

Supplement: Supplementary file 5 — Additional file 5: Figure S4. Arrangement of brain and ventral ganglia in various pycnogonid families. Optical sagittal sections; µCT scans. Where discernible, the white arrowheads point to the vestigial posterior ganglion anlagen at the dorso-posterior side of leg ganglion 4. A: Callipallene brevirostris (Callipallenidae), male. B: Ascorhynchus castellioides (Ascorhynchidae), male. C: Pallenopsis vanhoeffeni (Pallenopsidae), subadult. D: Ammothea clausi (Ammotheidae), male. E: Ammothea sp. (Ammotheidae), female. F: Ammothella biunguiculata (Ammotheidae), male. G: Ammothella longipes (Ammotheidae), female. [file 12983_2022_459_MOESM5_ESM.tif]

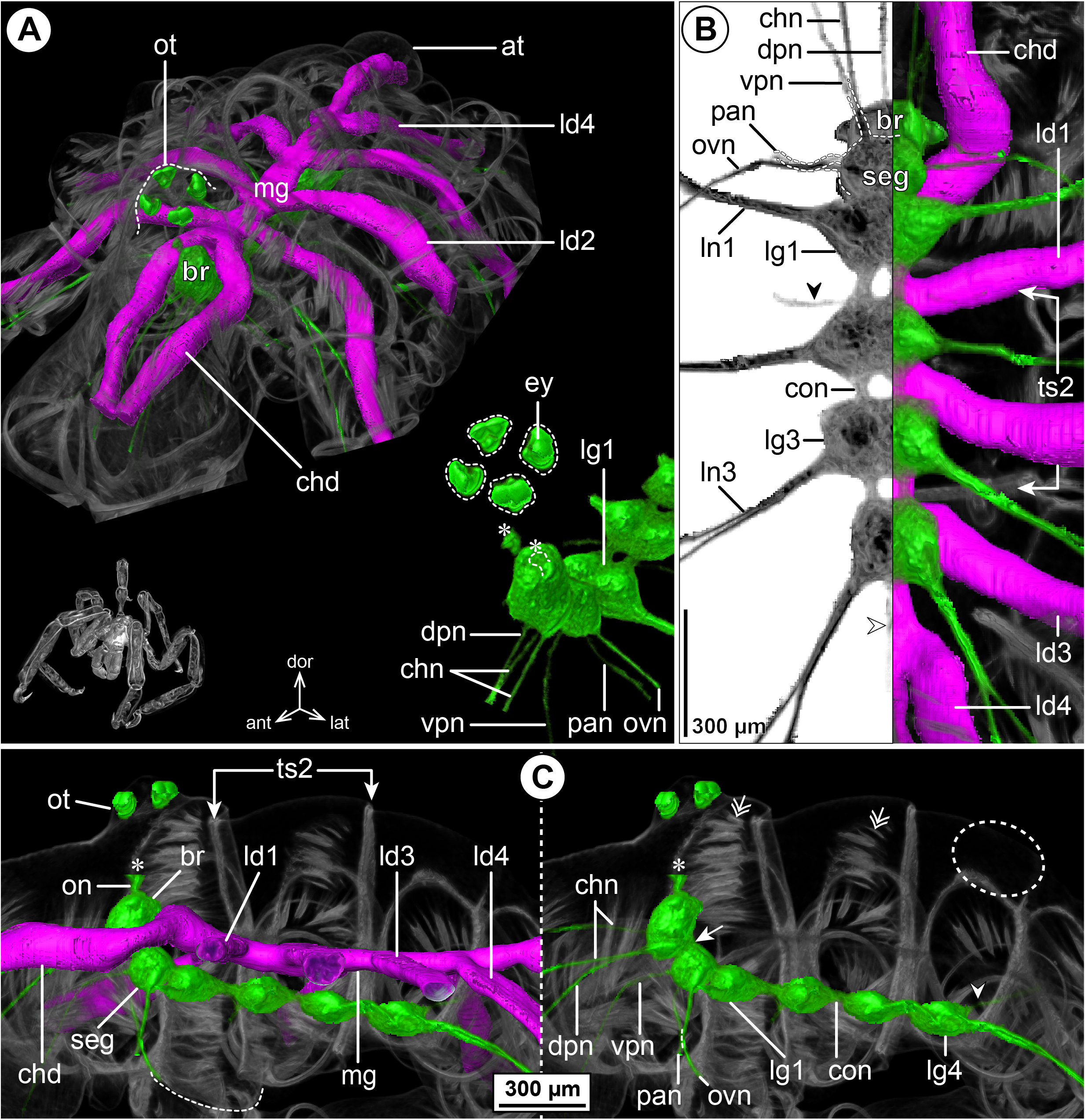

Supplement: Supplementary file 6 — Additional file 6: Figure S5. The central nervous system and midgut in the trunk of Stylopallene cheilorhynchus (Callipallenidae). Reconstructions of the CNS (3D volume rendering, green) and midgut (3D surface, magenta) based on a µCT scan of an adult male. The white arrowheads point to the origin of the posteriorly projecting proctodeal nerve. Asterisks indicate incompletely reconstructed portions of the optic nerve (insufficient resolution and tissue damage). For better pattern visualization, all major nerves of the right body half were virtually removed in A (right side) and C. A: Oblique antero-lateral view. Left bottom corner: overview of the complete specimen. Top: complete CNS and midgut reconstruction. Right bottom corner: anterior portion of the CNS and eyes. B: Ventral view. Left side: CNS in grayscale. The black arrowhead points to an intersegmental nerve. Right side: CNS and midgut. For reference, unreconstructed dorsal parts of the trunk are shown in transparent gray. C: Lateral view of the CNS with and without the midgut structures (left and right, respectively). The white arrow highlights the fusion area of brain and subesophageal ganglion. For further reference, unreconstructed parts of the right body half are shown in transparent gray. Double arrows indicate dorsal longitudinal musculature in the cephalosoma and trunk segment 2. In the fused trunk segments 3 and 4, longitudinal musculature is missing (stippled oval). [file 12983_2022_459_MOESM6_ESM.tif]

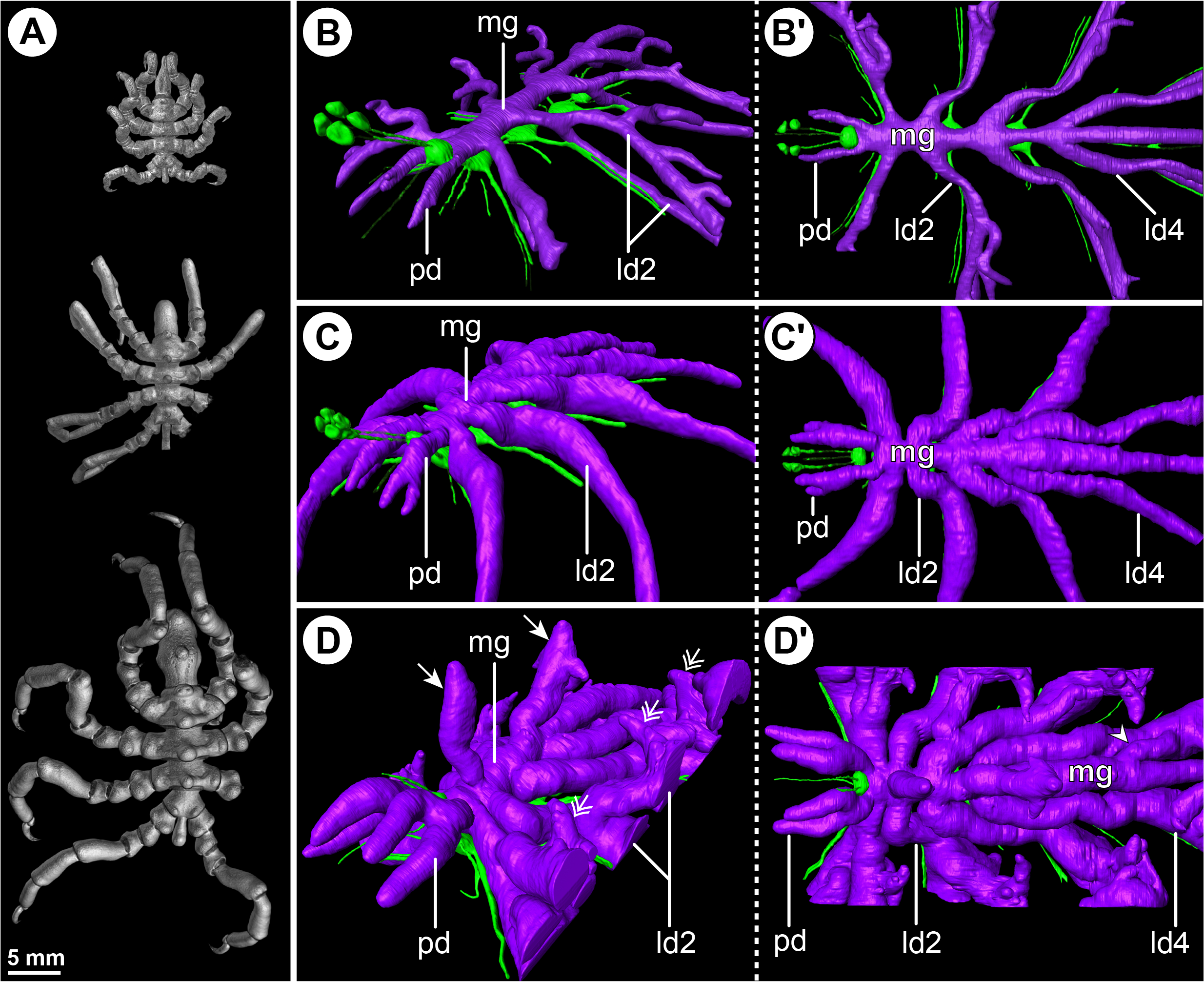

Supplement: Supplementary file 7 — Additional file 7: Figure S6. Comparison of the midgut branching pattern in the trunk of three species of the family Pycnogonidae. A: Volume renderings of µCT overview scans of complete specimens (from top to bottom: Pycnogonum litorale, P. gaini, P. diceros), dorsal view. Specimens are up to scale to illustrate the size differences. B–D’: 3D surfaces of CNS (green) and midgut (purple) reconstructions of the three species studied. For each species, an oblique antero-lateral view (left column) and a dorsal view (right column) is shown. B, B’: P. litorale. Note the widely spaced divergence points of the leg diverticula along the central midgut tube and their proximal bifurcation and further branching. C, C’: P. gaini. Note shorter distance between the leg diverticula’s divergence points and their simple linear structure. D, D’: P. diceros. Note closely spaced divergence points of the leg diverticula and their complex pattern of several sub-branches, including a dorsal projection that extends medially into the dorsal trunk (double arrowheads in D). Unpaired projections of the midgut extend into the dorso-median tubercles of the anterior trunk segments (arrows in D). Between the central gut tube and the right leg diverticulum 4, a non-bilaterally symmetrical secondary connection is present (arrowhead in D’). [file 12983_2022_459_MOESM7_ESM.tif]

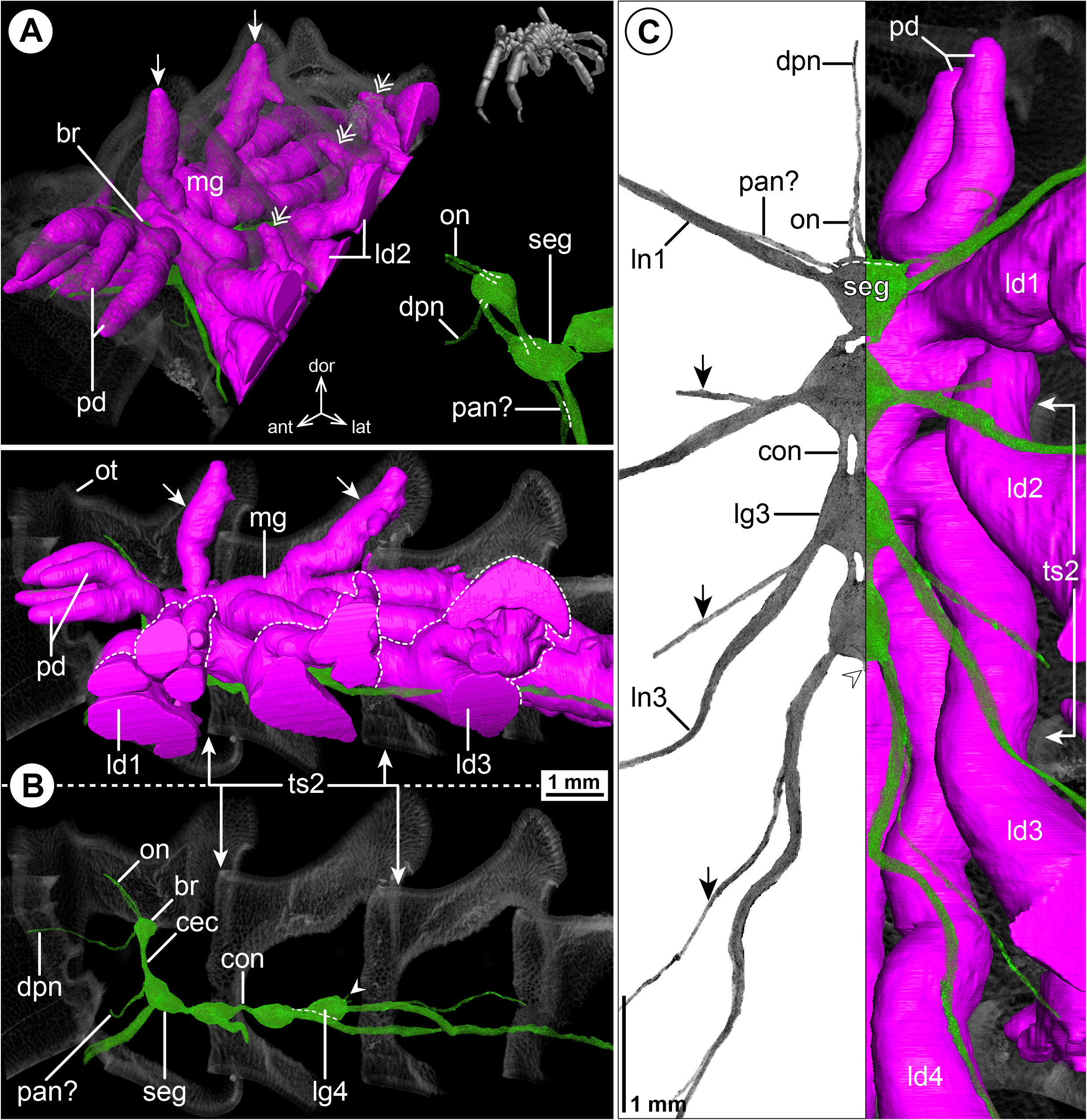

Supplement: Supplementary file 8 — Additional file 8: Figure S7. The central nervous system and midgut in the trunk of Pycnogonum diceros (Pycnogonidae). Reconstructions of the CNS (3D volume rendering, green) and midgut (3D surface, magenta) based on a µCT scan of an adult female. The white arrowheads point to the origin of the posteriorly projecting proctodeal nerve. White arrows indicate midgut projections into the dorso-median tubercles of the cephalosoma and of trunk segment 2. For better pattern visualization, all major nerves of the right body half were virtually removed in A (right side) and B. A: Oblique antero-lateral view. Right top corner: overview of the specimen. Left side: complete CNS and midgut reconstruction. The double arrowheads indicate projections from each leg’s dorsal diverticulum branch that extend dorso-medially into the trunk. Note that the voluminous proboscis diverticulum covers the view on the brain almost completely. Right bottom corner: anterior portion of the CNS. The eyes are not shown (not included in the µCT scan). B: Lateral view of the CNS with and without the midgut structures (top and bottom, respectively). The dorso-distal portions of the leg diverticula are highlighted (stippled lines) to illustrate their extensive branching in the trunk. For reference, unreconstructed parts of the right body half are shown in transparent gray. C: Ventral view. Left side: CNS in grayscale. Black arrows point to an additional branch of the leg nerves that extends to the body wall. Right side: CNS and midgut. For reference, unreconstructed dorsal parts of the trunk are shown in transparent gray. [file 12983_2022_459_MOESM8_ESM.tif]

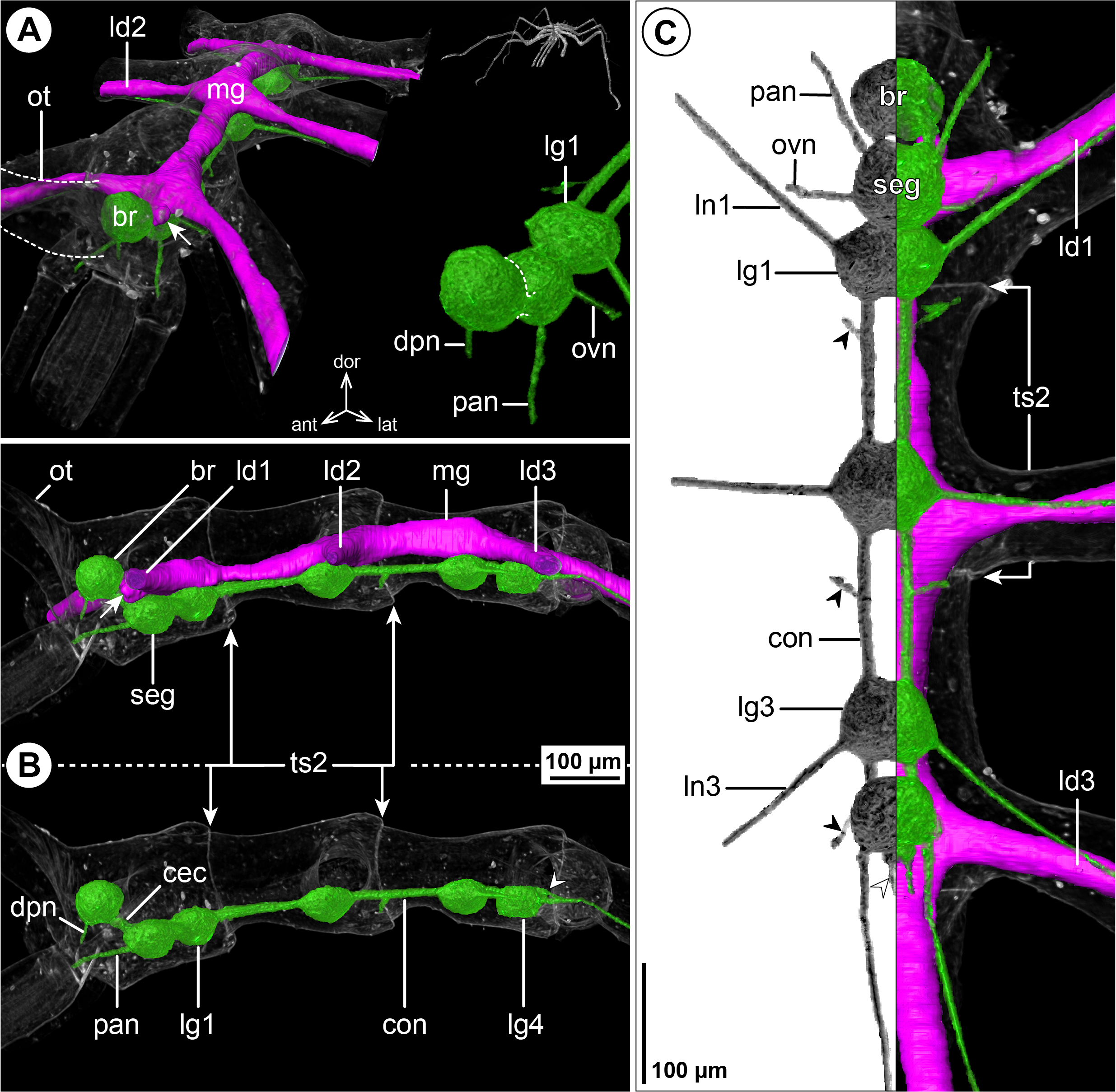

Supplement: Supplementary file 9 — Additional file 9: Figure S8. The central nervous system and midgut in the trunk of Pantopipetta armoricana (Austrodecidae). Reconstructions of the CNS (3D volume rendering, green) and midgut (3D surface, magenta) based on a µCT scan of a subadult. The white arrowheads indicate the origin of the posteriorly projecting proctodeal nerve. The white arrows indicate a small anterior midgut protrusion. Note that the eyes are not included in the scan, owing to the extreme elongation of the ocular tubercle. For better pattern visualization, all major nerves of the right body half were virtually removed in A (right side) and B. A: Oblique antero-lateral view. Right top corner: overview of the specimen. Left side: complete CNS and midgut reconstruction. Right bottom corner: anterior portion of the CNS and eyes. B: Lateral view of the CNS with and without the midgut structures (top and bottom, respectively). For reference, unreconstructed parts of the right body half are shown in transparent gray. C: Ventral view. Left side: CNS in grayscale. The black arrowheads point to the intersegmental nerves. Right side: CNS and midgut. For reference, unreconstructed dorsal parts of the trunk are shown in transparent gray. [file 12983_2022_459_MOESM9_ESM.tif]
